# RNA *N*^6^-methyladenosine reader protein YTHDF1 promotes plasma cell differentiation via IRF4 regulation in systemic lupus erythematosus

**DOI:** 10.1038/s12276-025-01587-x

**Published:** 2025-11-14

**Authors:** Shuang Lu, Xingyu Wei, Huan Zhu, Leyuan Li, Wenqian Zhang, Pei Du, Yaqin Yu, Meiling Zheng, Zhi Hu, Sujie Jia, Qianjin Lu, Ming Zhao

**Affiliations:** 1 Department of Dermatology, Human Key Laboratory of Medical Epigenomics, the Second Xiangya Hospital, Central South University, Changsha, China; 2 Hospital for Skin Diseases, Institute of Dermatology, Chinese Academy of Medical Sciences and Peking Union Medical College, Nanjing, China; 3 Key Laboratory of Basic and Translational Research on Immune-Mediated Skin Diseases, Chinese Academy of Medical Sciences, Nanjing, China; 4 Department of Pharmacy, Chinese Academy of Medical Sciences and Peking Union Medical College, Nanjing, China

**Keywords:** Rheumatoid arthritis, Epigenetics

## Abstract

B cell malfunction is implicated in the pathogenesis of systemic lupus erythematosus (SLE) through the release of proinflammatory cytokines and the production of autoreactive antibodies. RNA *N*^6^-methyladenosine (m^6^A) is the predominant post-transcriptional RNA modification that has been reported to control various biological processes. Whether RNA m^6^A alteration and m^6^A reader protein YTHDF1 contribute to B cell activation and terminal B cell differentiation in SLE has not been fully demonstrated. Here we observed that SLE peripheral B cell subsets, activated B cells and differentiated plasma cells (PCs) had abnormally elevated levels of YTHDF1, the deficit of which attenuated PC differentiation both in vitro and in mouse models that have been immunized with keyhole limpet hemocyanin (KLH) or *N*-propionyl polysialic acid (NP–KLH). Utilizing RNA sequencing, RNA immunoprecipitation, m^6^A immunoprecipitation and other functional experiments, we have identified and described a PC-promoting role of YTHDF1. YTHDF1 binds to the m^6^A-marked 3′ untranslated region of transcription factor IRF4 messenger RNA to enhance its stability, thereby facilitating PC differentiation. Depletion of YTHDF1 hindered the differentiation of PCs, reduced the generation of autoantibodies and ameliorated the lupus-like phenotypes in an imiquimod-treated mouse model. Overall, this study highlights a distinct role of YTHDF1 in promoting PC differentiation through the direct regulation of IRF4 in an m^6^A-dependent manner and identifies YTHDF1 as a potential target for the treatment of SLE.

## YTHDF1 drives plasma cell differentiation in lupus

Systemic lupus erythematosus (SLE) is a complex autoimmune disease in which the immune system attacks the body’s own tissues. Researchers have been exploring ways to manage SLE by targeting specific immune cells called B cells. This study investigates a protein called YTHDF1, which is involved in the regulation of B cells. They found that YTHDF1 levels are higher in certain B cells of patients with SLE. They conducted experiments using both human cells and mice to understand how YTHDF1 affects B cell behavior. They used techniques such as gene editing and RNA analysis to study the effects of removing YTHDF1 from B cells. This study showed that without YTHDF1, B cells had trouble developing into plasma cells, which are crucial for producing antibodies. The results suggest that YTHDF1 helps to stabilize a key molecule called IRF4, which is important for plasma cell development.

This summary was initially drafted using artificial intelligence, then revised and fact-checked by the author.

## Introduction

Systemic lupus erythematosus (SLE) is an intricate autoimmune disorder that is characterized by the malfunction of the immune system and inflammation-induced damage to multiple organs, in which the excessive activation of B cells and the differentiation of antibody-producing plasma cells (PCs) play an essential role^1,2^. Belimumab, a medicine recently approved by the Food and Drug Administration for the treatment of SLE, is a specific inhibitor that targets B lymphocyte stimulator (BLyS)^3,4^. Drugs or antibodies that specifically target the signaling pathways involved in B cell activation, such as CD40/CD40L and B-cell receptor, have shown considerable efficacy in the treatment of SLE^5,6^. These findings indicate the promising therapeutic potential of targeting B cells during their activation and differentiation for the management of SLE.

The differentiation of PCs is precisely controlled by transcriptional reprogramming and epigenetic mechanisms. High levels of the transcription factor IRF4 activate the transcription factor BLIMP1, which in turn controls the expression of genes needed for the differentiation of PCs^7,8^. Conversely, low levels of IRF4 activate the transcriptional repressor Bcl6, which promotes the fate of germinal center (GC) cells^9,10^. In addition, XBP-1 modifies PCs for antibody secretion by increasing the production of chaperones and other components associated with protein folding in the endoplasmic reticulum^11^.

RNA *N*^6^-methyadenosine (m^6^A) modification is one of most prevalent post-transcriptional modifications to influence mRNA splicing, translation, export, stability and decay^12^. The reversible m^6^A methylation is catalyzed by a methyltransferase complex comprising methyltransferase-like 3 (METTL3), METTL14 and Wilms tumor 1-associating protein (WTAP)^13,14^ and removed by demethylases obesity-associated protein (FTO) and AlkB homolog 5 (ALKBH5)^15,16^. The recognition and binding of m^6^A modification sites are predominantly mediated by YTH domain-containing family proteins (YTHDF1–3 and YTHDC1–2) and insulin-like growth factor 2 messenger RNA-binding proteins (IGF2BP1–3)^17^. The YTHDF proteins consist of three parts—the conserved YTH domain, the short carboxy terminus and the differential amino-terminal effector domain—with the variation in the N terminus determining the functional diversity of these proteins^18^. The YTHDF2 protein mainly mediates mRNA degradation^19,20^ and YTHDF1 could affects translation and degradation of target transcripts^21–24^, while YTHDF3 has been shown to regulate both transcript translation and stability^25,26^.

In recent years, RNA m^6^A modification has gained considerable interest in autoimmune reactions, including B cell autoimmunity. YTHDF2 has been shown to be required for GC formation^27^. METTL3 potentially inhibits B cell apoptosis and promotes B cell proliferation by regulating EZH2^28^. YTHDF1 was reported to be involved in IgG1 antibody production by targeting IGHG^29^. Another study proposed that YTHDF2 is required for the process of interleukin (IL)-7-induced differentiation of large pre-B cells from pro-B cells by binding to the m^6^A-marked mRNA and promoting their degradation. This study also demonstrated that the differentiation of small pre-B cells from large pre-B cells induced by IL-7 stimulation was independent of YTHDF1^30^.

Here we found that the expression of YTHDF1 was notably upregulated in SLE peripheral B subsets, activated B cells and differentiated PCs. The absence of YTHDF1 altered the distribution of distinct B cell subsets and significantly inhibited PC differentiation both in vitro and in vivo. Mechanistically, the depletion of *Ythdf1* compromised the differentiation of PCs by destabilizing m^6^A-marked IRF4 mRNA. The conditional knockout of *Ythdf1* in B cells resulted in a decrease in the generation of autoantibodies and an improvement in the lupus-like phenotypes in mice. These observations suggest that YTHDF1 contributes to the pathogenesis of SLE by regulating PC differentiation, making it a potential target for therapeutic interventions in SLE immunotherapy.

## Materials and methods

### Patients and healthy participants

We collected whole blood samples from patients with SLE from the Second Xiangya Hospital who satisfied the diagnostic criteria of the 2012 Systemic Lupus International Collaborating Clinics^31^ and from age- and gender-matched healthy controls (HCs) among the medical staff. Both patients and HCs provided informed consent on the basis of the use of ethical and safe protocols as approved by the Human Subjects Institutional Review Boards of the Second Xiangya Hospital, Central South University. The demographic and clinical characteristics of the patients and HCs are presented in Supplementary Table 1.

### In vitro B cell isolation and culture

Human and mouse CD19^+^ B cells or resting B cells were isolated using CD19 microbeads or CD43 microbeads (Miltenyi Biotec), respectively, from peripheral blood mononuclear cells. Cells were then cultured in Roswell Park Memorial Institute 1640 medium (Gibco) supplemented with 10% fetal bovine serum (Gibco), 1% penicillin–streptomycin (Beyotime) and β-mercaptoethanol (55 mM) at 37 °C with 5% CO_2_. Human B cells were stimulated with a combination of CpG (3·5 μg/ml, Invivogen) and anti-IgM F(ab′)_2_ (5 μg/ml, Jackson ImmunoResearch Laboratories). Mouse B cells were stimulated with a combination of lipopolysaccharides (LPS) (10 μg/ml), IL-4 (10 ng/ml) and IL-5 (10 ng/ml).

### siRNA transfection

Human B cells were transfected with *YTHDF1* small interfering (siRNA) or negative control siRNA (si-NC) using the P3 Primary Cell 4D Nucleofector Kit and Amaxa Nucleofector system (Lonza) according to the manufacturer’s protocols. In brief, B cells were collected and resuspended in 100 μl transfection reagent, and 10 μl siRNA (20 μM) was subsequently added to the cells and mixed thoroughly before electroporation. The siRNA sequences targeting *YTHDF1* are presented in Supplementary Table 2.

### RT–qPCR

Total RNAs were extracted from cells lysed by TRIzol (MRC), followed by complementary DNA synthesis with the PrimeScript RT Kit (Takara). Quantitative PCR with reverse transcription (RT–qPCR) reactions were performed with SYBR Green Master Mix (Bio-Rad) on a CFX96 Connect real-time system (Bio-Rad). Primer sequences are provided in Supplementary Table 2.

### Western blot

Isolated or cultured cells were collected using RIPA lysis buffer (Beyotime) supplemented with proteinase inhibitor cocktail (Roche). The protein concentrations were measured using the Pierce BCA Protein Assay Kit (Thermo Fisher Scientific). Protein samples were heated and separated on SDS–polyacrylamide gel electrophoresis (SDS–PAGE) and transferred onto PVDF membranes (Millipore). Following the blocking step with 5% nonfat milk, the membranes were subjected to overnight incubation with primary antibodies at 4 °C. Subsequently, the membranes were incubated with horseradish peroxidase-conjugated secondary antibodies for 1 h. Thereafter, the protein bands were visualized using SuperSignal West Pico PLUS (Thermo Fisher Scientific). Information on antibodies is presented in Supplementary Table 3.

### Mice and KLH or NP–KLH immunization

C57BL/6J mice were purchased from SLAC. The loxP–*Ythdf1*–loxP mice were constructed by Shanghai Biomodel Organism Science and Technology Development Company and bred with *Cd19-cre* mice. Eight-week-old *Ythdf1*-deficient mice and wild type (WT) mice were immunized subcutaneously with keyhole limpet hemocyanin (KLH; 2.5 mg/kg) (Sigma-Aldrich) emulsified in Complete Freund’s adjuvant (100 μl per injection) or *N*-propionyl polysialic acid (NP)–KLH (10 mg/kg) mixed with Alum (100 μl per injection). Blood samples were collected weekly from the submaxillary veins. All mice were killed after 28 days.

### RNA-seq

Splenic resting B cells in *Ythdf1*-deficient mice and WT mice were isolated and stimulated by IL-4, IL-5 and LPS for 3 days. Total RNAs were extracted and submitted to Novogene for RNA sequencing (RNA-seq). Genes with different expression levels between the two groups were filtered using a *P* value <0.05.

### IMQ-induced lupus-like model

Eight-week-old *Ythdf1*-deficient mice and WT control mice were topically administered with imiquimod (IMQ) cream (15 mg per mouse) three times per week on alternate days. Urine protein was detected using a colorimetric assay strip (URIT). Serum samples were obtained every 2 weeks. Mice were killed after 10 weeks.

### ELISA

Anti-mouse IgG, IgM (Thermo Fisher Scientific), and anti-double stranded DNA (anti-dsDNA) were detected following the manufacturer’s protocols. For NP-specific IgM and IgG measurements, high binding plates were coated with NP–BSA (5 μg/ml) overnight at 4 °C. The plates were blocked with 1% BSA for 1 h at room temperature. Diluted serum samples were subsequently introduced into precoated wells and incubated for 2 h at room temperature, followed by incubation with horseradish peroxidase-conjugated secondary antibodies (Bethyl Laboratories) for 1 h. 3,3′,5,5′-Tetramethylbenzidine substrate was added to each well and incubated for several minutes in the dark. Thereafter, the reactions were terminated and measured at a wavelength of 450 nm. Reagents and antibodies are presented in Supplementary Table 3.

### Histology

Tissues from mouse kidneys and draining lymph nodes (dLNs) were fixed in paraformaldehyde and embedded in paraffin. Kidney slices were subjected to hematoxylin–eosin (H&E) staining to evaluate the renal pathology. Multiplex immunohistochemistry was conducted using antibodies against IRF4 (CST), C3 (Abcam) and IgG (Abcam) following the standard protocol. After being incubated with the primary antibodies and secondary antibody, the slides were subjected to opal 7-Color Manual IHC Kit (Perkin Elmer) for fluorescence staining. Multilayer images were taken by Perkin Elmer and analyzed using the Mantra system. Information on antibodies is presented in Supplementary Table 3.

### Flow cytometry

Single-cell suspensions of lymphocytes from blood, spleen, dLNs or cultured B cells were incubated with fluorochrome-labeled antibodies for half an hour at 4 °C for surface staining. For intracellular staining, cells were surface stained and fixed using the Transcription Factor Buffer Set (BD Biosciences) for 1 h. Thereafter, the cells were washed and subsequently stained with antibodies targeting intercellular markers for an additional 30 min. Then the samples were analyzed on a BD flow cytometer (FACS Canto II, BD Biosciences). Information on antibodies used is presented in Supplementary Table 4.

### RNA m^6^A quantification

The global m^6^A level of the samples was detected using the EpiQuick m^6^A RNA Methylation Quantification kit (Colorimetric) (EpigenTek). In brief, 200 ng RNA of each sample was coated to the wells. The capture antibody was used on each well after a washing step, followed by the stepwise addition of detection antibody, enhancer solution and color-developing solution. The m^6^A concentrations were calculated by measuring the absorbance of each well at a wavelength of 450 nm on the basis of the standard curve.

### MeRIP

m^6^A immunoprecipitation (MeRIP) was performed using the EpiQuik CUT&RUN m^6^A RNA Enrichment (MeRIP) kit (EpigenTek) according to the instructions issued by the manufacturer. In brief, the purified mRNAs were digested and fragmented using RNA fragmentation reagent. The anti-m^6^A antibody was preincubated with beads in IP buffer at room temperature for 1 h. Fragment mRNAs were subsequently added to the antibody-beads mixture and subjected to overnight incubation with rotation at 4 °C. The immunoprecipitated mixture was digested by proteinase K to release the captured RNAs, which were then extracted using the phenol–chloroform extraction procedures. The m^6^A modifications were predicted by sequence-based RNA adenosine methylation site predictor analysis (http://www.cuilab.cn/sramp)^32,33^. Respective primer sequences are presented in Supplementary Table 2.

### RIP

An RNA immunoprecipitation (RIP) assay was conducted using the Magna RIP Kit (Millipore) in accordance with the instructions provided by the manufacturer. In brief, 5 μg anti-YTHDF1 and anti-rabbit IgG antibodies were separately incubated with 50 μl magnetic beads. The cell lysates were prepared and incubated overnight with the antibody–bead mixture and rotation at 4 °C. Subsequently, the immunoprecipitated complexes were digested with proteinase K to free the RNAs. The RNAs that were extracted using the phenol–chloroform method then underwent standard RT–qPCR analysis. The enrichment of RNAs in each sample was compared to the percentage of RNA in the input sample.

### Luciferase reporter assay

The IRF4 coding sequence (CDS) or 3′ untranslated region (3′UTR) segments (~500 bp) were amplified and inserted into the dual-luciferase pmirGLO vector (Promega). The Quick-Change II Site-Directed Mutagenesis kit (Agilent Technologies) was used to produce mutations from A to T at validated m^6^A-tagged sites. The dual-luciferase vector and mouse *Ythdf1-*Flag plasmids were cotransfected into HEK293T cells using Lipofectamine 3000 reagent (Invitrogen). Luciferase activity was quantified using the Dual-Luciferase Reporter Assay System (Promega). The activity of the firefly luciferase was standardized by dividing it by the activity of the Renilla luciferase.

### Ribosome profiling and analysis

Extraction of the ribosome-protected fraction, library preparation and high-throughput sequencing were performed by CloudSeq by following the manual for the GenSeq Ribo Profile kit (GenSeq). In brief, the cycloheximide-treated cells were lysed with lysis buffer and digested with nuclease. Digested samples were purified with size exclusion chromatography to obtain ribosome footprints. Ribosome-protected fragments were size-selected with PAGE and subjected to ribosomal RNA removal. Then, purified RNA fragments were end-repaired, ligated with 3′ adapters and reverse-transcribed to cDNA. The cDNA was PAGE-gel purified, circularized and amplified with PCR. The resulting libraries were purified and sequenced in a NovaSeq platform (Illumina).

### RNA degradation assay

CD19^+^ B lymphocytes derived from *Ythdf1*-deficient mice and control mice were separated and stimulated for 12 h. Actinomycin D (Sigma-Aldrich) was added to each sample at a final concentration of 10 μM, and the cells were collected at time points 0, 30, 60 and 90 min.

### Protein stability

Mouse splenic B cells were stimulated for 24 h, followed by the treatment of 100 μg/ml cycloheximide (CHX) (MCE) for 3, 6, and 9 hours. Thereafter, cells were collected and subjected to western blot analysis to determine the IRF4 protein level.

### Statistical analysis

Data analyses were performed using GraphPad Prism software. A two-tailed Student’s *t*-test was used to compare the statistical difference between two groups. Analysis of variance (ANOVA) models were applied to compare the differences among several groups. The Pearson correlation analysis was used to examine the correlation between the groups. Results are presented as mean ± s.d., unless otherwise noted. The statistical significance between distinct groups was calculated as described in each figure.

## Results

### YTHDF1 expression changes in peripheral B cells of patients with SLE

**Fig. 1:**
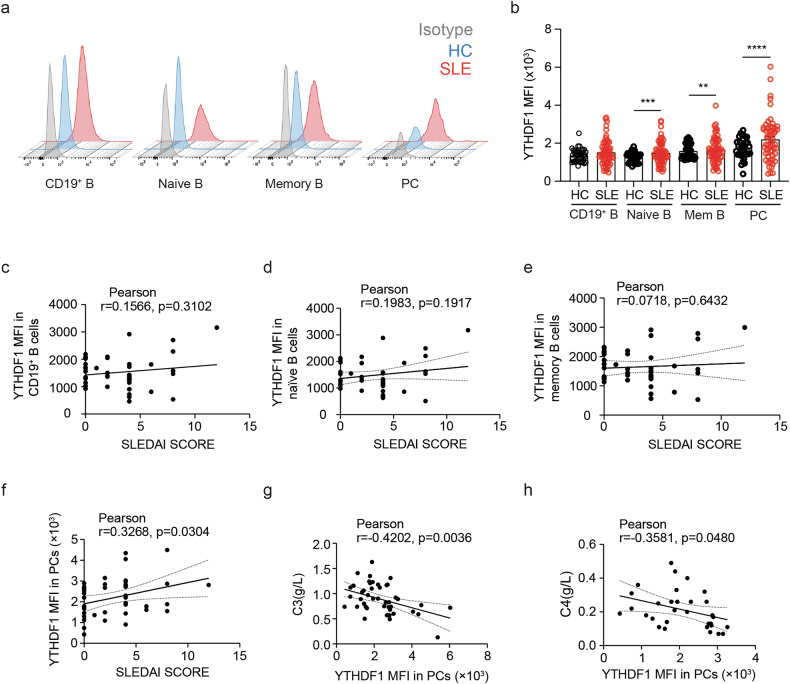
YTHDF1 expression changes in SLE peripheral B cells. **a** Flow cytometry histograms depicting the expression of YTHDF1 in peripheral CD19^+^ B cells, naive B cells, memory B cells and PCs in patients with SLE relative to HCs. Naive B, CD19^+^CD20^+^IgD^+^CD27^−^ cells; memory B, CD19^+^CD20^+^IgD^−^CD27^+^ cells; PC, CD19^+^CD20^−^CD38^+^ cells. **b** Mean fluorescence intensity (MFI) of YTHDF1 expression in each B cell subset. HC, *n* = 41; SLE, *n* = 58. **c**–**f** Pearson correlation analysis between SLEDAI score and MFI of YTHDF1 expression in SLE peripheral CD19^+^ B cells (**c**), naive B cells (**d**), memory B cells (**e**) and PCs (**f**). SLEDAI: SLE disease activity, *n* = 48. **g**, **h** Pearson correlation analysis between the MFI of YTHDF1 expression in SLE PCs and the levels of C3 (**g**) (*n* = 46) and C4 (**h**) (*n* = 31) in SLE serum. ^**^P < 0.01, ^***^P < 0.001, ^****^P < 0.0001. Unpaired two-tailed Student’s *t*-test (**b**) or Pearson’s correlation analysis (**c**–**h**).

To investigate the potential role of YTHDF proteins in B cell homeostasis in SLE, we first collected peripheral blood mononuclear cells from both HCs and patients with SLE. Flow cytometry analysis indicated that the YTHDF1 level in CD19^+^ B cells was comparable but was significantly elevated in SLE-naive B cells, memory B cells and PCs compared to HCs (Fig. 1a, b and Supplementary Fig. 1a). Compared to HCs, YTHDF2 and YTHDF3 proteins were higher in SLE CD19^+^ B cells, naive B cells and memory B cells but were similar in PCs (Supplementary Fig. 1b,c). Given the notion of PCs as effective B cells that secrete the predominant cytokines and autoantibodies, we sought to investigate the involvement of YTHDF1 in B cell function. Further analysis indicated no notable correlation between SLE disease activity and *YTHDF1* expression in CD19^+^ B cells (Fig. 1c), naive B cells (Fig. 1d) or memory B cells (Fig. 1e). However, a significantly positive correlation was observed between the expression of *YTHDF1* in PCs and SLE disease activity (Fig. 1f). In addition, inverse correlations between the expression of *YTHDF1* in PCs and the levels of complement 3 (C3) (Fig. 1g) and C4 (Fig. 1h) were identified. These data indicate the potential involvement of YTHDF1 in controlling aberrant differentiation of PCs in SLE.

### YTHDF1 is required for PC differentiation

**Fig. 2:**
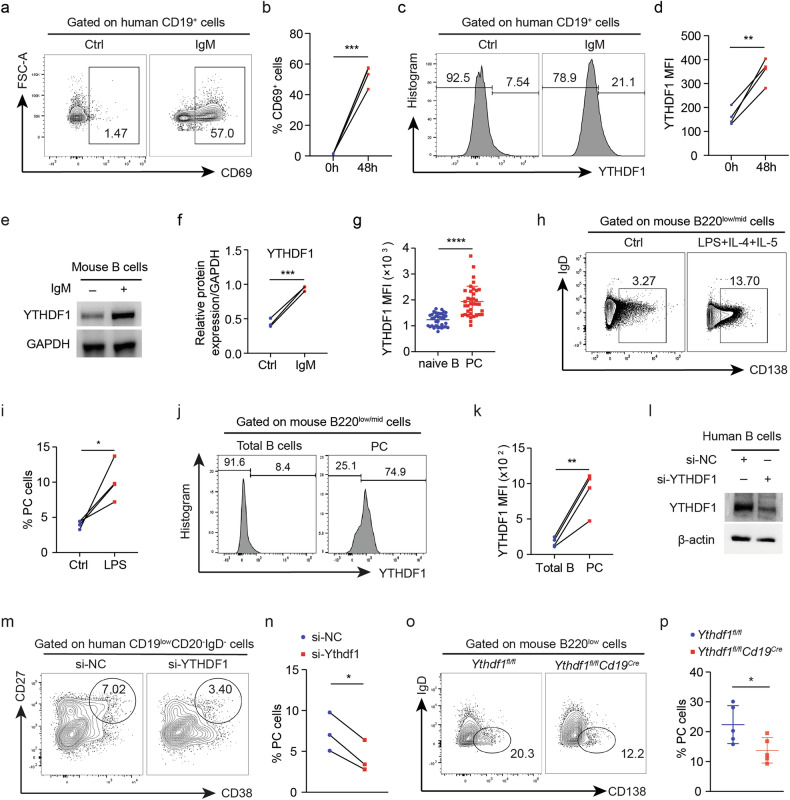
YTHDF1 is required for PC differentiation. **a** Representative dot blots showing the proportions of human peripheral CD19^+^ B cells treated and activated by IL-4 and IgM and control cells. **b** Quantification of CD19^+^CD69^+^ activated B cells, *n* = 4. **c** Flow histogram showing the relative YTHDF1 expression in human peripheral CD19^+^ B cells treated and activated by IL-4 and IgM and control cells. **d** Quantification of YTHDF1 MFI, *n* = 4. **e** Western blot analysis comparing the YTHDF1 protein levels between mice splenic resting B cells activated by IL-4 and IgM and control cells. Glyceraldehyde-3-phosphate dehydrogenase (GAPDH) was used as a loading control. **f** Quantification of YTHDF1 protein levels, *n* = 3. **g** Flow analysis detecting the expression of YTHDF1 in peripheral naive B cells and PCs from HCs, *n* = 36. **h** Representative dot blots showing the proportions of mouse splenic B220^low/mid^ cells treated with IL-4, IL-5 and LPS compared to control cells. **i** Quantification of B220^low/mid^IgD^−^CD138^+^ PCs, *n* = 4. **j** Flow histogram showing the relative YTHDF1 expression in mouse splenic B cells and in vitro-differentiated PCs. **k** Quantification of YTHDF1 MFI, *n* = 4. **l** Western blot detecting human B cells treated with either negative siRNA or YTHDF1 siRNA when PC differentiation was induced in vitro. β-actin was used as a loading control. **m** Representative dot plots showing the proportions of CD19^low^CD20^−^IgD^−^ cells in human B cells treated with either YTHDF1 siRNA or si-NC. **n** Quantification of CD19^low^CD20^−^IgD^−^CD27^+^CD38^+^ PCs, *n* = 3. **o** Representative dot plots showing the proportions of splenic CD43^−^ B cells isolated from *Ythdf1*^*fl/fl*^*Cd19*^*Cre*^ mice and *Ythdf1*^*fl/fl*^ control mice after being stimulated by IL-4, IL-5 and LPS for 5 days. **p** Quantification of B220^low^IgD^−^CD138^+^ cells, *n* = 5. ^*^P < 0.05, ^**^P < 0.01, ^***^P < 0.001, ^****^P < 0.0001. Paired two-tailed Student’s *t*-test (**b**, **d**, **f**, **i**, **k** and **n**) or unpaired two-tailed Student’s *t*-test (**g** and **p**).

We then examined the expression of YTHDF1 in activated B cells and effector B cells. Activated B cells from both humans (Fig. 2a–d) and mice (Fig. 2e, f and Supplementary Fig. 2a–b) exhibited increased levels of YTHDF1 relative to their resting B cell counterparts. Substantial increases in YTHDF1 protein levels were confirmed in peripheral PCs (Fig. 2g) and mouse differentiated PCs (Fig. 2h–k). These data therefore collectively indicate the involvement of YTHDF1 in B cell activation and PC differentiation.

We next examined the constant involvement of YTHDF1 in the differentiation of human PCs by suppressing its expression using siRNA (Fig. 2l). In addition to a reduced activated B cells (Supplementary Fig. 2c, d), flow cytometry analysis demonstrated a decrease in the population of PCs after *YTHDF1* knockdown (Fig. 2m, n). We utilized the CRISPR–Cas9 method to create *Ythdf1*^flox/flox^ mice, which were then bred with *Cd19*^Cre^ mice to generate *Ythdf1*^flox/flox^*Cd19*^Cre^ offsprings with specific depletion of *Ythdf1* in *Cd19*^+^ B cells (Supplementary Fig. 2e, f). Deletion of *Ythdf1* in B cells had no impact on B cell proliferation (Supplementary Fig. 3a, b), activation (Supplementary Fig. 3c,d) and early apoptosis (Supplementary Fig. 3e, f) but slightly suppressed the late apoptosis of B cells (Supplementary Fig. 3e, g). The analysis of B cell subset distribution revealed that, except for increased GC B cells in the dLNs of *Ythdf1*-deficient mice (Supplementary Fig. 4a, e, f), the percentages and absolute numbers of naive B cells (Supplementary Fig. 4b, g, h), memory B cells (Supplementary Fig. 4c, i, j) and PCs (Supplementary Fig. 4d, k, l) in the spleen and dLNs were similar between *Ythdf1*-deficient mice and WT control mice. These data suggest that the absence of *Ythdf1* has minimal effects on B cell equilibrium and specialization in the steady state. Next, we isolated splenic resting B cells and induced their differentiation in vitro. It was observed that B cells lacking *Ythdf1* displayed an attenuated differentiation of PCs compared to their control littermates (Fig. 2o, p and Supplementary Fig. 1d), suggesting that the conditional removal of *Ythdf1* impairs the forced differentiation of PCs.

### YTHDF1 deficiency inhibits PC differentiation and antibody secretion

**Fig. 3:**
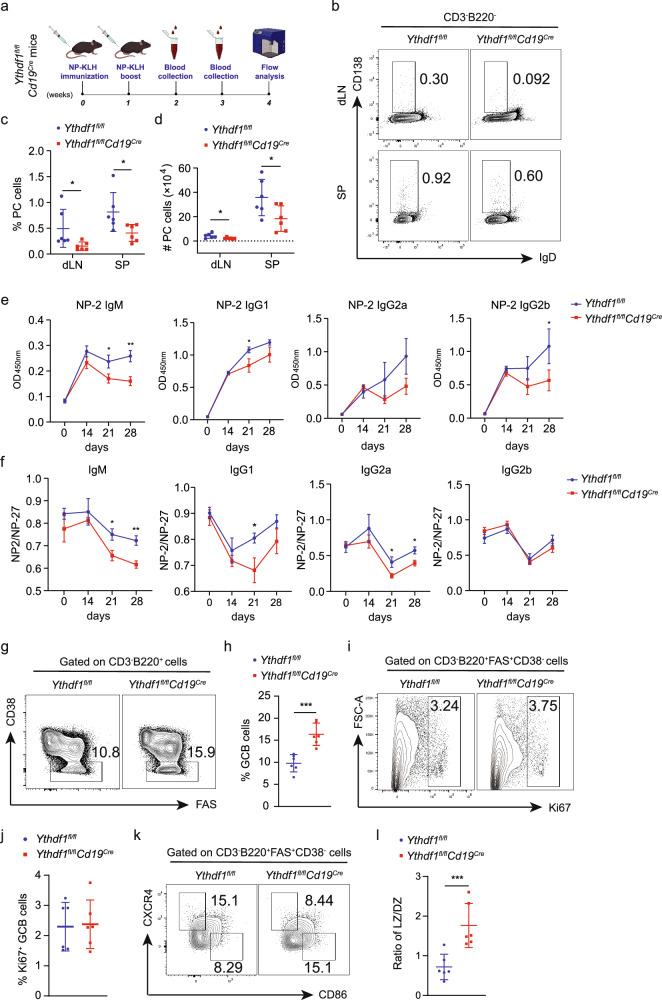
YTHDF1 deficiency inhibits B cell-mediated humoral immune responses in vivo. **a** A schematic of the NP–KLH-immunized mouse model. **b** Representative dot plots showing the proportions of CD3^−^B220^−^ cells in the dLNs and spleen (SP) of NP–KLH-immunized WT and *Ythdf1*-deficient mice. **c**, **d** Quantification of PC frequency (**c**) and absolute number (**d**) in the spleen and dLNs of WT and *Ythdf1*-deficient mice after NP–KLH challenge, *n* = 6. **e** ELISA of NP-2-specific IgM, IgG1, IgG2a and IgG2b antibodies in the serum of NP–KLH-immunized WT and *Ythdf1*-deficient mice (mean ± s.e.m.). **f** NP-27-specific IgM, IgG1, IgG2a and IgG2b antibodies in the serum of NP–KLH-immunized WT and *Ythdf1*-deficient mice were determined using ELISA (mean ± s.e.m.). **g** Representative dot plots showing the proportions of CD3^−^B220^+^ cells in the spleen of NP–KLH-immunized Ythdf1-deficient mice and WT mice. **h** Quantification of the frequency of CD3^−^B220^+^Fas^+^CD38^−^ GC B cells, *n* = 6. **i** Representative dot plots showing the proportions of CD3^−^B220^+^Fas^+^CD38^−^ cells in the spleen of NP−KLH-immunized Ythdf1-deficient mice and WT mice. **j** Quantification of the frequency of CD3^−^B220^+^Fas^+^CD38^−^Ki67^+^ cells, *n* = 6. **k** Representative dot plots showing the proportions of CD3^−^B220^+^Fas^+^CD38^−^ cells in the spleen of NP–KLH-immunized Ythdf1-deficient mice and WT mice. **l** The ratio of LZ (CD86^hi^CXCR4^lo^) to DZ (CD86^lo^CXCR4^hi^) GC B cells in the spleen of NP–KLH-immunized Ythdf1-deficient mice and WT mice, *n* = 6. ^*^P < 0.05, ^**^P < 0.01, ^***^P < 0.001. Two-way ANOVA with Sidak’s multiple comparisons test (**c** and **d**) or unpaired two-tailed Student’s *t*-test (**e**, **f**, **h**, **j** and **l**).

To investigate whether YTHDF1 is required for B cell-mediated humoral immune responses, *Ythdf1*-deficient mice and their WT littermates were immunized with KLH (Supplementary Fig. 5a) or NP–KLH (Fig. 3a). No noticeable distinctions were seen between WT and *Ythdf1*-deficient mice in terms of body weight (Supplementary Fig. 5b), spleen weight (Supplementary Fig. 5c), total B cells (Supplementary Fig. 5d, e) and activated B cells (Supplementary Fig. 5f, g) in the spleen and dLNs after KLH immunization. Nevertheless, we observed a decline in the quantity of PCs in the dLNs, a reduction in both the proportion and absolute number of PCs in the spleen (Supplementary Fig. 5h–j) and an increase in the frequency of GC B cells in both the spleen and dLNs (Supplementary Fig. 5k–m). The impaired differentiation of PCs was accompanied by a decrease in the production of IgM at an earlier time and a reduced IgG level at the later stage (Supplementary Fig. 5n, o).

The absence of *Ythdf1* did not affect the mice body weight (Supplementary Fig. 6a), spleen weight (Supplementary Fig. 6b) or total B cells (Supplementary Fig. 6c,d) after NP–KLH challenge. Notably, *Ythdf1*-deficient mice displayed a decrease in the number of activated B cells (Supplementary Fig. 6e,f) and a lower percentages and absolute numbers of PCs in the spleen and dLNs (Fig. 3b–d) compared to their control counterparts. The defective differentiation of PCs led to a notable decrease in the levels of high-affinity (NP-2) IgM antibodies and IgG antibodies (Fig. 3e, f). The decrease in the ratio of NP-2 to NP-27 antibody titers in *Ythdf1*-deficient mice after NP–KLH immunization suggests an impairment in antibody affinity maturation (Fig. 3f). A consistent increase in GC B cells isolated from the spleen other than dLNs was observed after *Ythdf1* depletion, indicating a potential disturbance to the GC reaction (Fig. 3g, h and Supplementary Fig. 6g,h). The accumulation of GC B cells in *Ythdf1*-deficient GCs was not likely owing to increased cell proliferation, as indicated by comparable Ki67-positive cells between Ythdf1-deficient GC B cells and the WT counterparts (Fig. 3i, j). Besides proliferation in the dark zone (DZ), GC B cell selection in the light zone (LZ) and migration between the DZ and LZ are also essential for antibody affinity maturation. Further analysis of GCs revealed that cells of the LZ phenotype and the ratio of the frequencies of LZ to DZ cells were significantly increased in *Ythdf1*-deficient splenic GCs (Fig. 3k, l and Supplementary Fig. 6i, j), suggesting a conceivable interruption of B cell selection and transition from LZ to DZ to complete the GC cycles for affinity maturation. The evaluation of IgG1^+^ and IgG3^+^ cells yielded a consistent reduction in IgG-specific PCs and uptrend in IgG3^+^ GC B cells (Supplementary Fig. 6k–n). These findings, together with the observations in the KLH-immunized model, collectively indicate that *Ythdf1* deficiency impairs PC differentiation and antibody secretion.

### YTHDF1 depletion ameliorates the lupus-like phenotype in mice

**Fig. 4:**
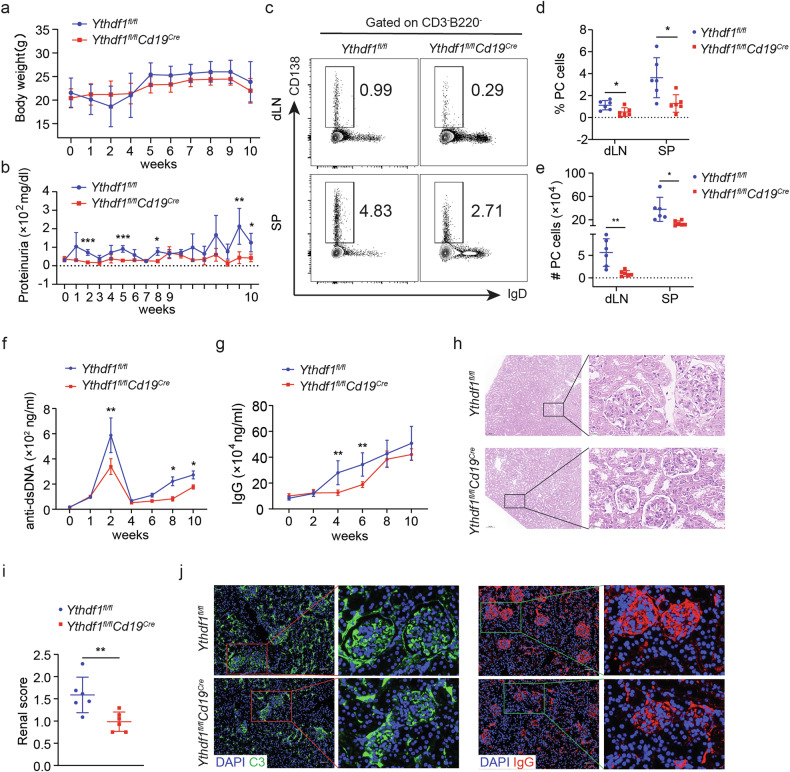
YTHDF1 depletion ameliorates the lupus-like phenotypes in mice. **a** The body weights of *Ythdf1*-deficient mice and WT control mice treated ectopically with IMQ. **b** The proteinuria levels of *Ythdf1*-deficient mice compared to WT control mice after IMQ administration. **c** Representative dot plots showing the proportions of CD3^−^B220^+^ cells in the spleen and dLNs of IMQ-treated mice. **d**, **e** Quantification of PC frequency (**d**) and absolute number (**e**) in the spleen and dLNs of IMQ-treated mice. **f**, **g** ELISA comparing the levels of dsDNA antibody (**f**), and total IgG antibody (**g**) in the serum of *Ythdf1*-deficient mice and WT control mice following IMQ treatment (mean ± s.e.m.). **h** Representative H&E staining images of kidney sections from *Ythdf1*-deficient mice and WT control mice after IMQ treatment. Scale bar, 100 μm (left) and 20 μm (right). **i** Calculation of the average grade of glomerular lesions for *Ythdf1*-deficient mice and WT control mice after IMQ treatment. **j** Representative C3-stained (left) and IgG-stained (right) glomeruli of IMQ-treated mice. Scale bar, 50 μm. ^*^P < 0.05, ^**^P < 0.01, ^***^P < 0.001. Unpaired two-tailed Student’s *t*-test (**a**, **b**, **f**, **g** and **i**) or two-way ANOVA with Sidak’s multiple comparisons test (**d** and **e**).

To induce a mouse model that mimics lupus, we topically administered IMQ to both WT mice and *Ythdf1*-deficient mice as previously described^34–36^. Mice treated with IMQ exhibited characteristics similar to lupus-like conditions, such as noticeable splenomegaly (Supplementary Fig. 7a). The absence of *Ythdf1* did not affect the mice body weight (Fig. 4a) or spleen weight (Supplementary Fig. 7b) but it significantly reduced proteinuria at weeks 2, 5, 8 and 10 after IMQ treatment (Fig. 4b). Flow analysis of single cell suspensions at 10 weeks revealed similar levels of total B cells (Supplementary Fig. 7c,d) and activated B cells (Supplementary Fig. 7e,f). Similarly, it showed a significantly greater quantity of splenic GC B cells (Supplementary Fig. 7g,h) and a lower frequency and absolute count of PCs in both the spleen and dLNs of *Ythdf1*-deficient mice (Fig. 4c–e), indicating the blockade of PC differentiation. By flow analysis, both the proportion of GCs of LZ phenotype and the ratio of LZ to DZ were increased in *Ythdf1*-deficient mice (Supplementary Fig. 7i,j). Immunohistochemistry staining revealed an increased LZ area (Supplementary Fig. 7k) and LZ to DZ ratio (Supplementary Fig. 7l), a comparable proportion of Ki67-positive cells (Supplementary Fig. 7m,o) and enhanced cleaved caspase3 staining in DZ cells (Supplementary Fig. 7n,o). These observations further support the impairment of GC B cell selection and migration after *Ythdf1* depletion, which could cause defects in antibody affinity maturation as seen in humoral immune mice. In line with a reduced proportion of PCs, the production of anti-dsDNA (Fig. 4f) and total IgG (Fig. 4g) antibodies was subsequently decreased in mice lacking *Ythdf1*. *Ythdf1* deficiency also yielded less severe renal damage and fewer depositions of C3 and IgG in the kidney glomerulus, as indicated by H&E staining (Fig. 4h, i) and histological analysis (Fig. 4j), respectively. These observations collectively suggest that the absence of *Ythdf1* inhibits the differentiation of PCs, reduces the production of autoantibodies and ameliorates the lupus-like phenotypes in mice.

### YTHDF1 regulates PC-associated genes and signaling

**Fig. 5:**
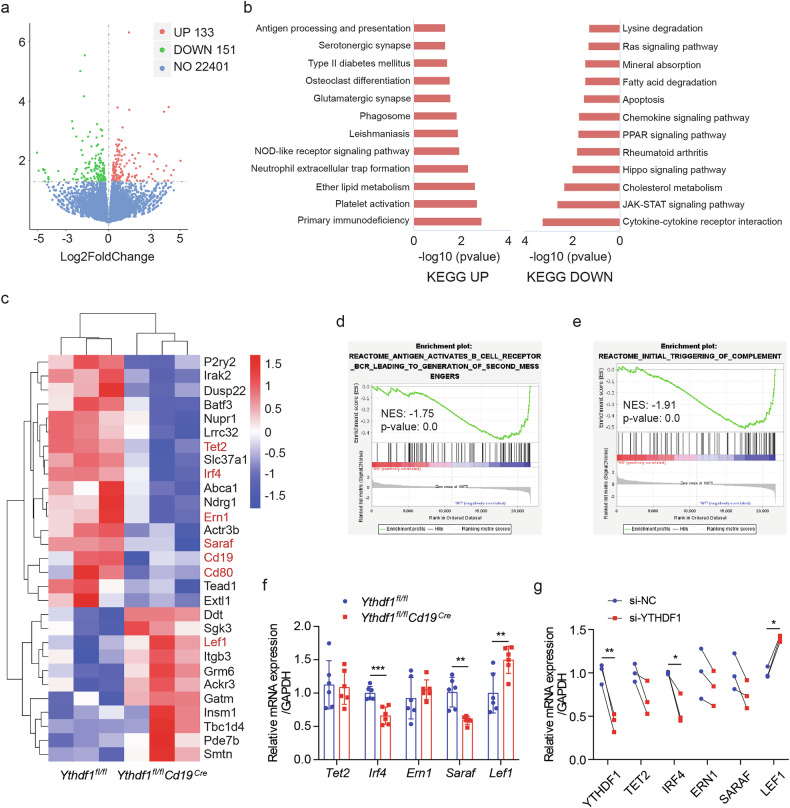
YTHDF1 regulates PC-associated genes and signaling. **a** Volcano plot of genes with differential expression levels in splenic B cells treated with IL-4, IL-5 and LPS (*Ythdf1*^*fl/fl*^*Cd19*^*Cre*^ versus WT), *n* = 3. **b** Kyoto Encyclopedia of Genes and Genomes (KEGG) analysis of differentially expressed genes (*Ythdf1*^*fl/fl*^*Cd19*^*Cre*^ versus WT). **c** A heat map showing gene expression level in splenic B cells treated with IL-4, IL-5 and LPS (*Ythdf1*^*fl/fl*^*Cd19*^*Cre*^ versus WT). **d**, **e** Gene set enrichment analysis of differentially expressed genes (*Ythdf1*^*fl/fl*^*Cd19*^*Cre*^ versus WT). **d**, B cell receptor signaling; **e**, triggering of complement. **f** RT–qPCR comparing mRNA expression of genes between splenic B cells in *Ythdf1*^*fl/fl*^*Cd19*^*Cre*^ and control mice after KLH immunization, *n* = 6. **g** RT–qPCR comparing mRNA expression of genes between human stimulated B cells treated with either YTHDF1 siRNA or si-NC, *n* = 3. ^*^P < 0.05, ^**^P < 0.01, ^***^P < 0.001. Two-way ANOVA with Sidak’s multiple comparisons test (**f**) or paired two-tailed Student’s *t*-test (**g**).

To demonstrate the fundamental mechanism by which YTHDF1 regulates PC differentiation, we performed RNA-seq in stimulated mouse splenic B cells with or without *Ythdf1* depletion. A total of 284 significantly altered genes in *Ythdf1*-deficient cells were identified, among which 133 were upregulated and 151 were downregulated (Fig. 5a). Further analysis suggested that genes suppressed by *Ythdf1* depletion were more closely correlated with PC differentiation. Downregulated genes were significantly enriched in the JAK–STAT pathway^37^ and Ras signaling pathway^38^ (Fig. 5b), which was associated with the differentiation of antibody-secreting cells. In addition, *Ythdf1* deficiency resulted in significantly reduced expression of transcripts *Tet2*^39,40^, *Irf4*^9^, *Ern1*^41,42^ and *Saraf*^43^, which were recognized to facilitate PC differentiation. By contrast, a PC-inhibiting gene *Lef1*^44^ was increased in *Ythdf1*-deficient cells (Fig. 5c). Gene set enrichment analysis also revealed that downregulated genes enriched in B cell receptor signaling (Fig. 5d) and triggered the complement (Fig. 5e), in support of the regulatory role of *Ythdf1* in B cell function and immune responses. Next, we conducted RT–qPCR analysis to confirm the consistent downregulation of *Irf4* and its downstream target *Prdm1* and the upregulation of *Lef1* in both *Ythdf1*-null mouse B cells (Fig. 5f) and *YTHDF1*-knockdown human B cells (Fig. 5g).

**Fig. 6:**
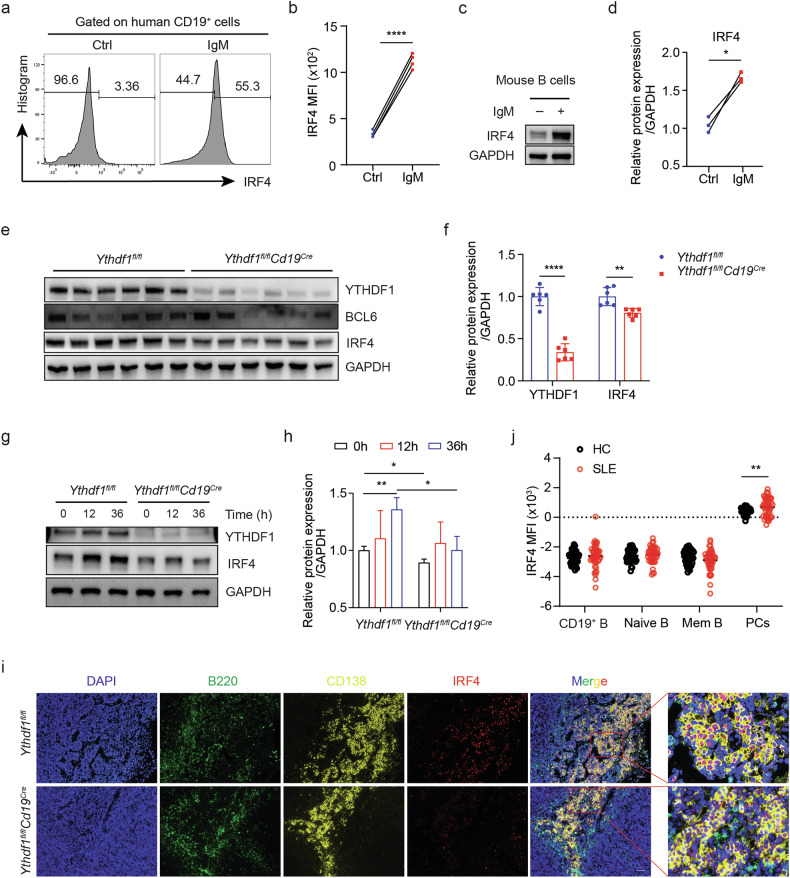
YTHDF1 regulates IRF4 expression during PC differentiation. **a** A flow histogram showing the relative IRF4 expression in human peripheral CD19^+^ B cells treated and activated by IL-4 and IgM and control cells. **b** Quantification of YTHDF1 MFI, *n* = 4. **c** The protein levels of IRF4 in WT splenic resting B cells stimulated by IL-4 and IgM relative to control cells were determined by western blot analysis. GAPDH was used as a loading control. **d** Quantification of IRF4 protein levels, *n* = 3. **e** The protein levels of YTHDF1, BCL-6 and IRF4 in splenic B cells in both WT and *Ythdf1*-deficient mice after KLH immunization were measured by western blot analysis. GAPDH was used as a loading control. **f** Quantification of protein levels, *n* = 6. **g** The protein expression of YTHDF1 and IRF4 in stimulated splenic B cells from WT and *Ythdf1*^flox/flox^*Cd19*^Cre^ mice. GAPDH was used as a loading reference. **h** Quantification of IRF4 protein levels, *n* = 3. **i** Representative histological images comparing IRF4 expression in lymph node tissues of *Ythdf1*-deficient mice and WT control mice. Scale bar, 50 μm. **j** MFI of IRF4 expression in each B cell subset. HC, *n* = 44; SLE, *n* = 45. ^*^P < 0.05, ^**^P < 0.01, ^****^P < 0.0001. Paired two-tailed Student’s *t*-test (**b** and **d**), two-way ANOVA with Sidak’s multiple comparisons test (**f**), two-way ANOVA with Tukey’s multiple comparisons test (**h**) or unpaired two-tailed Student’s *t*-test (**i**).

Among the differentially expressed genes, *Irf4* was particularly intriguing owing to its indispensable role in PC differentiation. We first confirmed that in vitro-stimulated human and mouse B cells had notably increased amounts of IRF4 (Fig. 6a–d), the expression of which was significantly decreased following *Ythdf1* depletion (Fig. 6e, f). The notable upregulation of IRF4 when inducing PC differentiation was largely compromised when *Ythdf1* was depleted (Fig. 6g, h). Furthermore, the histological examination of lymph node tissues in KLH-immunized mice (Fig. 6i) and lupus mice (Supplementary Fig. 7p) also indicated that IRF4 was predominantly expressed in CD138^+^ PCs, an expression pattern that was apparently diminished in the absence of *Ythdf1*. In line with these observations in mice, IRF4 was also principally expressed in human PCs, the level of which was much higher in SLE peripheral PCs than in HCs (Fig. 6j). Collectively, these results indicate that YTHDF1 facilitates the differentiation of SLE PCs, potentially by regulating the transcription factor IRF4.

### YTHDF1 binds to m^6^A-tagged 3′UTR of *Irf4* mRNA to control its stability

**Fig. 7:**
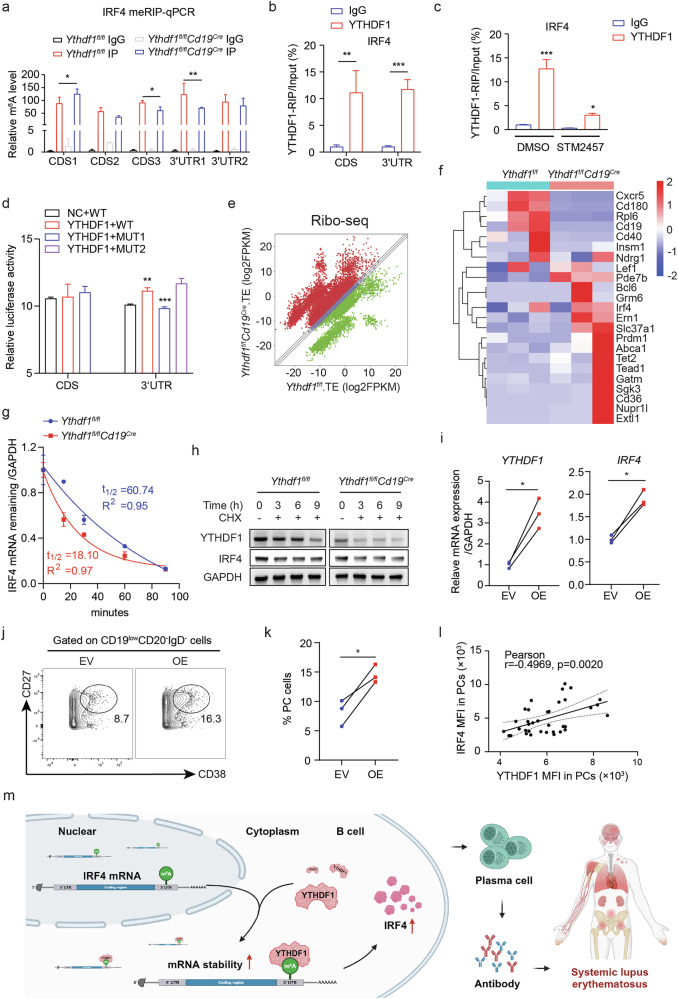
YTHDF1 binds to m^6^A-tagged 3′UTR of *Irf4* mRNA to control its stability. **a** MeRIP–qPCR comparing the m^6^A enrichment on five predicted m^6^A sites of *Irf4* mRNA between splenic B cells from *Ythdf1*-null mice and WT control mice. The results are presented as a relative percentage normalized to inputs, *n* = 3. **b** RIP–qPCR analysis evaluating the binding of YTHDF1 to *Irf4* mRNA in splenic B cells of WT mice, *n* = 3. **c** RIP–qPCR analysis comparing the binding of YTHDF1 to *Irf4* mRNA in WT mice splenic B cells treated with STM2457 or dimethylsulfoxide (DMSO). STM2457, a specific catalytic inhibitor of m^6^A methyltransferase METTL3, *n* = 3. **d** The relative luciferase activity of WT and mutant vectors of *Irf4* CDS or 3′UTR sequence that were cotransfected with either an empty vector (EV) or a YTHDF1 overexpression vector into HEK293T cells, *n* = 4. **e** A scatter plot of TE changes in splenic B cells treated with IL-4, IL-5 and LPS (Ythdf1fl/flCd19Cre versus WT). TE was calculated by dividing the abundance of the ribosome-protected fragments by transcript abundance from the RNA-seq input, *n* = 3. **f** A heat map showing the relative level of mRNA bound to the ribosome in splenic B cells treated with IL-4, IL-5 and LPS (Ythdf1fl/flCd19Cre versus WT). **g** RT–qPCR analysis detecting the relative remaining *Irf4* mRNA in WT and *Ythdf1*-deficient mice after treatment with actinomycin D for the indicated time period, *n* = 3. **h** Western blot analysis of IRF4 protein level upon CHX treatment for the indicated times in splenic B cells from *Ythdf1*-deficient mice and control mice, *n* = 3. The splenic B cells were prestimulated with LPS for 24 h before CHX treatment to amplify IRF4 expression. **i** RT–qPCR analysis detecting the expression of *YTHDF1* and *IRF4* mRNA in human B cells transfected with EV or YTHDF1 overexpression plasmid (OE) when PC differentiation was induced in vitro, *n* = 3. **j** Representative dot plots showing the proportions of CD19^low^CD20^−^IgD^−^ cells in human B cells transfected with EV or YTHDF1 OE when PC differentiation was induced in vitro. **k** Quantification of CD19^low^CD20^−^IgD^−^CD27^+^CD38^+^ PCs, *n* = 3. **l** Pearson correlation analysis between the MFI of YTHDF1 expression in SLE PCs and IRF4 expression in PCs (*n* = 36). **m** Schematic of the proposed model elucidating the regulatory role of YTHDF1 in the differentiation of PCs during the development of SLE. ^*^P < 0.05, ^**^P < 0.01, ^***^P < 0.001. Two-way ANOVA with Tukey’s multiple comparisons test (**a** and **d**), two-way ANOVA with Sidak’s multiple comparisons test (**b** and **c**), Michaelis–Menten model (**g**), paired two-tailed Student’s *t*-test (**h** and **k**) or Pearson’s correlation analysis (**l**).

Given its function as an m^6^A reader protein, YTHDF1 binds to m^6^A-modified mRNAs to determine their fate^17^. To examine the mechanism by which YTHDF1 regulates IRF4 expression, we predicted the presence of m^6^A modifications on *Irf4* mRNA. The sequence-based RNA adenosine methylation site predictor analysis identified five m^6^A sites on *Irf4* mRNA with high confidence or very high confidence, three of which were located in the CDS region and the remaining two which were found on the 3′UTR (Supplementary Fig. 8a). MeRIP–qPCR analysis confirmed that these sites were indeed labeled with m^6^A. The modification abundance of some sites changed after *Ythdf1* removal, indicating the potential involvement of writers or erasers that mediate m^6^A modifications in a *Ythdf1*-dependent manner (Fig. 7a). In support of the regulatory role of *Ythdf1* on *Irf4*, a direct interaction between YTHDF1 and *Irf4* mRNA was identified using RIP–qPCR with primers targeting either the CDS or 3′UTR (Fig. 7b), which was dramatically diminished following the inhibition of m^6^A methyltransferase METTL3 (Fig. 7c). These findings additionally support that the interaction between YTHDF1 and *Irf4* mRNA depends on m^6^A modification.

To identify the potential binding site of YTHDF1 on *Irf4* mRNA, we cloned the CDS3 or 3′UTR sequence, as well as the mutant form of them by substituting A with T in the core motif (RRACH) of the identified m^6^A sites, into a dual-luciferase reporter construct (Supplementary Fig. 8b). The vectors were simultaneously transfected with either an empty vector or a *Ythdf1* construct into HEK293T cells. Comparable levels of luciferase activity were observed in cells transfected with vectors containing either the WT or mutant form of the CDS3 sequence. Nevertheless, the luciferase activity of cells transfected with the WT 3′UTR was seen to increase with overexpressed *Ythdf1*. This increase was eliminated by the mutant form of 3′UTR1 (m^6^A_5140_), rather than the 3′UTR2 (m^6^A_5036_), suggesting that the 3′UTR1 (m^6^A_5140_) of *Irf4* mRNA is required for the binding of YTHDF1 (Fig. 7d). To investigate how *Ythdf1* regulates *Irf4* expression, we first performed ribosome profiling (Ribo-seq) to compare the binding of mRNA to ribosomes between *Ythdf1*-deficient B cells and control cells. The number of transcripts that had elevated translational efficiency (TE) was higher than those in which TE was suppressed (Fig. 7e). The analysis of individual genes revealed that the binding of ribosomes to the mRNAs of *Cd19* and *Cxcr5* was significantly reduced after *Ythdf1* depletion, while the occupancies of ribosomes on mRNAs of *Irf4*, *Lef1*, *Ern1*, *Bcl6* and *Prdm1* remained largely unaffected (Fig. 7f), suggesting that *Ythdf1* deficiency does not contribute to *Irf4* translation. As both the mRNA level and the protein level of IRF4 declined upon *Ythdf1* depletion, we speculated that YTHDF1 could possibly regulate either the mRNA stability or the protein stability of IRF4. To this end, we treated both *Ythdf1*-deficient B cells and control B cells with actinomycin D. The degradation of *Irf4* mRNA was significantly accelerated after *Ythdf1* depletion, indicating that YTHDF1 controlled *Irf4* mRNA stability (Fig. 7g). Treating splenic B cells with protein synthesis inhibitor CHX suggested that the stability of IRF4 protein was not affected by *Ythdf1* loss (Fig. 7h and Supplementary Fig. 8c). Collectively, these results indicate that *Ythdf1* promotes *Irf4* expression by enhancing the stability of its mRNA via the recognition of the m^6^A-tagged 3′UTR region, thereby facilitating PC differentiation. Owing to the inherent difficulty in transfecting mouse B cells either via plasmid or lentiviral delivery, we overexpressed *YTHDF1* in human B cells through electroporation (Fig. 7i). We observed that forced expression of *YTHDF1* in human B cells markedly promoted *IRF4* expression (Fig. 7i) and PC differentiation (Fig. 7j, k). The regulatory role of YTHDF1 on IRF4 was further supported by an apparent positive correlation of their expression in SLE peripheral PCs (Fig. 7l).

## Discussion

In recent years, RNA m^6^A modification has emerged as an essential epitranscriptomic mechanism for controlling the translation and stability of certain transcripts. Here, we describe the role of m^6^A reader protein YTHDF1 in PC differentiation and the pathophysiology of SLE. Our findings demonstrate a notable upregulation of YTHDF1 expression in activated B cells and differentiated PCs. The depletion of YTHDF1 profoundly impaired PC differentiation and reduced antibody production both in vitro and in vivo, suggesting that YTHDF1 is required for PC differentiation. Moreover, the level of YTHDF1 expression remains consistently elevated in the peripheral B cells of patients with SLE, and its expression in PCs is correlated with higher SLE disease activity and reduced amounts of complements in the blood. Mechanistically, we propose that *Ythdf1* identifies and binds to m^6^A-tagged *Irf4* mRNA, thereby enhancing its stability to facilitate PC differentiation. This hypothesis is further supported by mouse experiments demonstrating that the deficiency of *Ythdf1* substantially impedes the expression of *Irf4*, diminishes the proportion of PCs and ameliorates the lupus-like phenotypes. Of note, a recent study by Wang et al. consistently demonstrated elevated levels of YTHDF1 in the antibody-secreting cells of patients with SLE and reduced autoantibody production upon YTHDF1 inhibition. This study and our research elucidate the function of YTHDF1 in SLE B cells from distinct perspectives, which provides complementary mechanistic insights and collectively supports the aggravative role of YTHDF1 in SLE pathogenesis.

Despite the extensive data showing that the aberrant differentiation of PCs with self-reactive properties is indispensable in several autoimmune diseases, including SLE, our comprehension of the regulatory mechanisms that govern PC differentiation remains incomplete. IRF4 is a critical transcription factor in determining the PC fate. Prior research has uncovered various mechanisms that affect PC differentiation through the regulation of IRF4. Elevated CD45 phosphatase activity in memory B cells enhances PC differentiation by facilitating the efficient activation of IRF4 and BLIMP1^45^, while FOXP1 inhibits PC differentiation by directly repressing the expression of IRF4, BLIMP1 and XBP1^46^. Although the regulation of IRF4 at the transcriptional level has been well examined, the involvement of post-transcriptional mechanisms in controlling IRF4 expression during PC differentiation has hardly been investigated. Here, we elucidate an RNA m^6^A-dependent regulatory mechanism of IRF4 by YTHDF1 in PC differentiation. Our findings not only shed light on the previously unidentified function of YTHDF1 in regulating B cell differentiation but also extend our knowledge of the epigenetic patterns that control B cell-mediated humoral responses. Notably, *Ythdf1*-deficient mice presented with elevated proportions of GC B cells, accumulation of LZ cells and enhanced apoptosis of DZ cells, which collectively indicated the impairment of B cell selection and migration, which are responsible for the defect in antibody affinity maturation. The reduced B cell activation observed in vitro upon *YTHDF1* knockdown was not reproduced in particular in vivo tests, indicating that the suppressive impact of *YTHDF1* on B cell activation could probably be offset by intricate internal regulatory systems.

YTHDF1–3 share an N-terminal low-complexity sequence and a conserved C-terminal YTH domain, suggesting the possibility of similarity in their target preference and functions. An investigation of the transcriptome-wide sequencing utilizing cross-linking and immunoprecipitation-based methods suggested that DF1, DF2 and DF3 exhibit identical binding patterns at m^6^A sites within mRNAs^47^. Another study conducted on HIV-1 additionally revealed that all DF proteins enhance mRNA stability and subsequently elevate protein expression by attaching to m^6^A sites located in the 3′UTR^48^.

The functional consequences of m^6^A modification mediated by YTHDF1 are probably determined by the cellular context, specific targets and the location of m^6^A. YTHDF1 has been discovered to bind m^6^A sites located near the stop codon of p65 mRNA to enhance its protein translation^49^. YTHDF1 could bind to E2F8 mRNA and enhance its mRNA stability^50^. Furthermore, YTHDF1 was demonstrated to promote the stability of BECN1 mRNA via recognizing the m^6^A binding site within coding regions^51^. Our data suggest that the m^6^A modification in the 3′UTR of *Irf4* mRNA recognized by YTHDF1 could enhance its stability. Of note, the m^6^A levels of certain sites in *Irf4* mRNA were altered in the absence of *Ythdf1*, which is not expected to be a direct consequence of *Ythdf1* depletion, as YTHDF1 functions as a reader protein. Research has shown that the nuclear YTHDF2 compete with the demethylase FTO to prevent the demethylation process of targeted transcripts during heat shock stress^52^. Thus, we suspect that the changes in m^6^A levels after depleting *Ythdf1* may be attributed to the actions of writers or erasers responsible for m^6^A modifications on *Irf4* mRNA, which could somehow be affected by the presence status of YTHDF1. Nevertheless, further investigation is needed to fully comprehend the functional interactions and potential regulatory networks among these proteins.

Despite the insights provided by our study on the YTHDF1–IRF4 axis in SLE, several limitations should be considered. The first one is the exclusive examination of YTHDF1 depletion on IRF4 expression and PC differentiation without considering functional similarities or differences among distinct YTHDF proteins. Appreciating the synergy and specificity of YTHDF proteins in B cell function in the future will be of interest to comprehensively reveal the m^6^A regulatory network in the pathogenesis of SLE. Second, on the basis of our results and the literature, YTHDF1 probably participates in B cell differentiation and functions through additional mechanisms beyond the one we have discussed, which require further exploration. Exploring these alternative possibilities will provide further evidence regarding the role of m^6^A modification in regulating antibody-mediated immune responses and its implications in autoimmunity. Third, the observed alterations following YTHDF1 knockout—including the shifted ratio between LZ and DZ cells, differential apoptosis rates in DZ and reduced antibody affinity—collectively suggest that YTHDF1 plays a critical role in GC reactions, somatic hypermutation and affinity maturation. However, the precise molecular mechanisms underlying these phenotypes remain to be fully elucidated. Future studies focusing on how YTHDF1 regulates GC B cell selection and migration will be essential to further unravel the m^6^A-related mechanisms governing humoral immune dysregulation and may ultimately reveal novel therapeutic targets for autoimmune diseases.

In summary, our work establishes a novel link between mRNA methylation and humoral immune dysregulation in SLE (Fig. 7m). The consistent pattern of reduced PC differentiation observed both in vitro and in living organisms, immunoglobins, anti-dsDNA antibody titles, proteinuria and glomerular deposition of immune complex in *Ythdf1*-deficient mice substantiates its significance in the development of SLE and suggests its potential as a promising target for SLE treatment.

## Supplementary information

Supplementary Information
