# Supplementary material for: RNA N6-methyladenosine reader protein YTHDF1 promotes plasma cell differentiation via IRF4 regulation in systemic lupus erythematosus
Source: Exp Mol Med. 2025 Nov 14;57(11):2574–87. doi: 10.1038/s12276-025-01587-x (PMC12686027; doi:10.1038/s12276-025-01587-x)
Supplement: Supplementary file 1 — Supplementary Information [file 12276_2025_1587_MOESM1_ESM.pdf]

RNA N6-methyladenosine reader protein YTHDF1 promotes plasma cell differentiation through the regulation of IRF4 in systemic lupus erythematosus

Dr. Shuang Lu[1#], Mr. Xingyu Wei[2, 3#], Ms. Huan Zhu[1], Ms. Leyuan Li[1], Ms. Wenqian Zhang[1], Dr. Pei Du[1], Ms. Yaqin Yu[1], Dr. Meiling Zheng[2, 3], Ms. Zhi Hu[2, 3], Professor. Sujie Jia[4], Professor. Qianjin Lu[1, 2, 3*], Professor. Ming Zhao[1, 2, 3*]

Supplementary materials

Supplementary Fig. 1 1
Supplementary Fig. 2 2
Supplementary Fig. 3 3
Supplementary Fig. 4 4
Supplementary Fig. 5 6
Supplementary Fig. 6 8
Supplementary Fig. 7 9
Supplementary Fig. 8 10
Supplementary Table 1. Information on HCs and patients 12
Supplementary Table 2. Sequence information on siRNA and primers 16
Supplementary Table 3. Information on antibodies for western blot, ELISA, and histology 18
Supplementary Table 4. Reagents for flow cytometry analysis 18

Supplementary Fig. 1

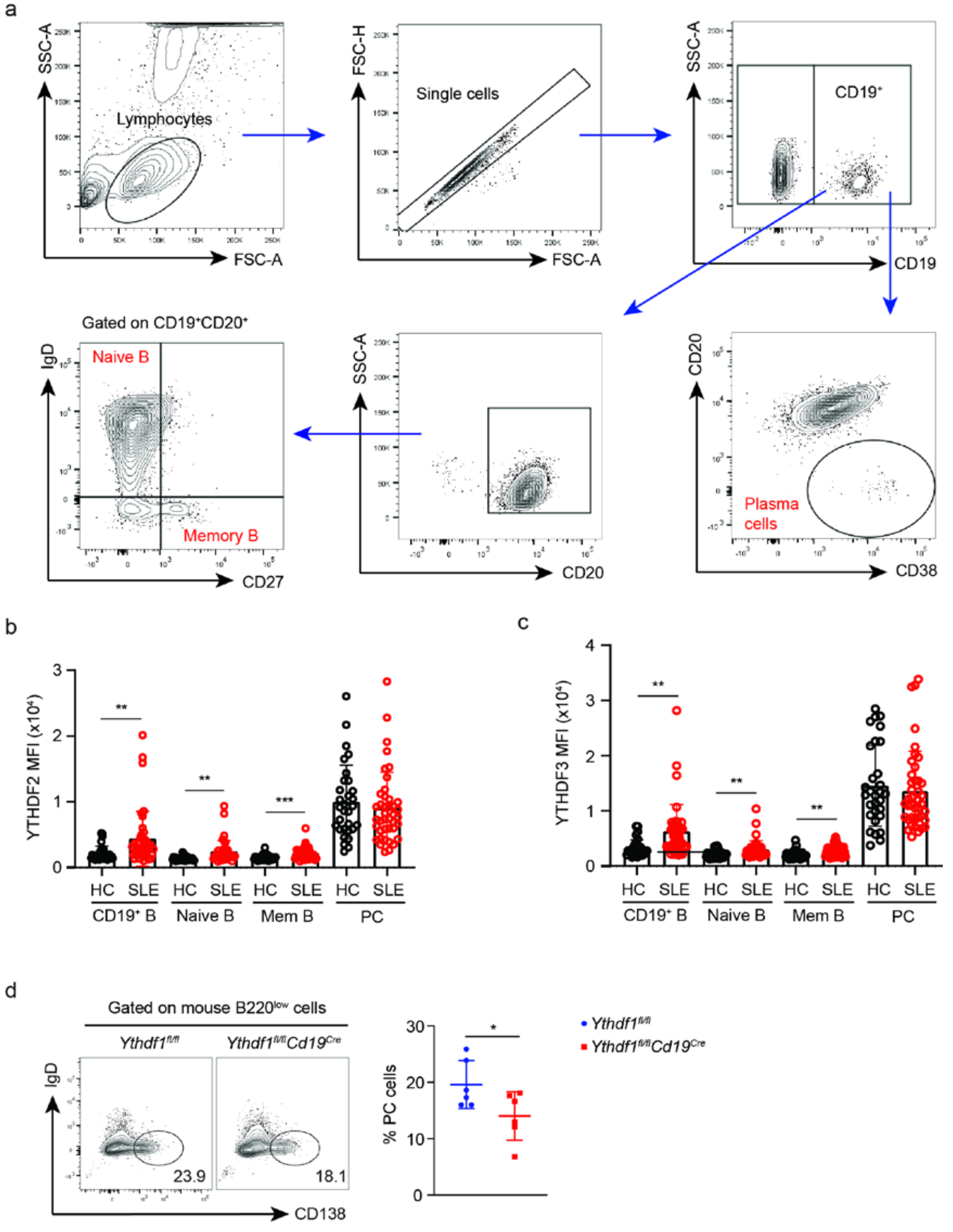

Supplementary Fig. 1 YTHDF1 expression in human B cells. a Gating strategies of B cell subsets in human PBMCs. Naive B cells: $CD19^{+}CD20^{+}IgD^{+}CD27^{-}$ cells; memory B cells: $CD19^{+}CD20^{+}IgD^{-}CD27^{+}$ cells; plasma cells: $CD19^{+}CD20^{-}CD38^{+}$ cells. **b** MFI of YTHDF2 expression in each B cell subset. HC: n=33; SLE: n=43. **c** MFI of YTHDF3 expression in each B cell subset. HC: n=33; SLE: n=43. **d** Representative dot plots showing the proportions of splenic $CD19^{+}$ B cells isolated from *Ythdf1*$^{fl/fl}$*Cd19*^{Cre} mice and *Ythdf1*$^{fl/fl}$ control mice after being stimulated by IL-4, IL-5, and LPS for 5 days. Quantification of $B220^{low}IgD^{-}CD138^{+}$ cells, n=6. *p<0.05, **p<0.01, ***p<0.001. (b-d) Unpaired two-tailed Student's *t* test.

Supplementary Fig. 2

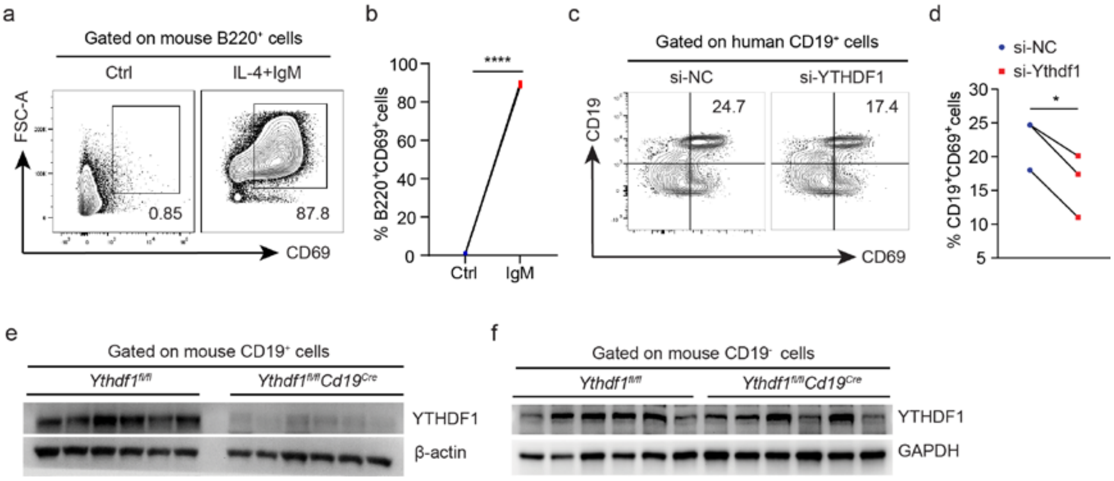

Supplementary Fig. 2 YTHDF1 expression is elevated in human activated B cells. a Representative dot plots showing the proportions of mouse B220^{+} cells stimulated with IL-4 and IgM compared with the control cells. **b** Quantification of B220^{+}CD69^{+} B cells, n=3. **c** Representative dot plots illustrating the proportions of human B cells treated with either negative siRNA or *YTHDF1* siRNA. **d** Quantification of CD19^{+}CD69^{+} activated B cells, n=3. **e-f** Western blot analysis comparing the YTHDF1 expression between *Ythdf1*-deficient mice and WT control mice in CD19^{+} cells (e) and CD19^{-} cells (f), β-actin and GAPDH were used as loading controls. *p<0.05, ****p<0.0001. (b, d) Paired two-tailed Student's *t* test.

Supplementary Fig. 3

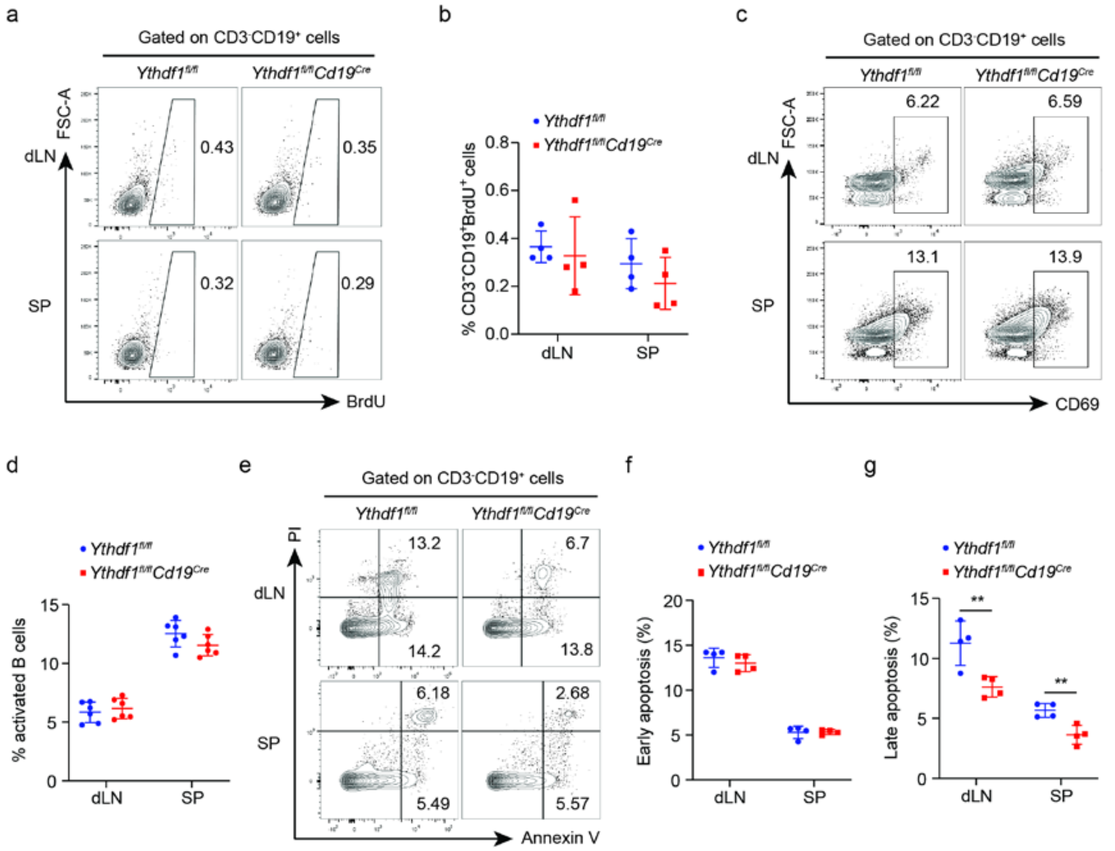

Supplementary Fig. 3 YTHDF1 is dispensable for B cell proliferation, activation, and early apoptosis in mice in the steady state. a, **c**, **e** Representative dot plots showing the proportions of CD3⁻CD19⁺ cells in the spleen and dLNs of *Ythdf1*-deficient mice and WT control mice. **b**, **d**, **f**, **g** Quantification of CD3⁻CD19⁺BrdU⁺ proliferating B cells (b), CD3⁻CD19⁺CD69⁺ activated B cells (d), CD3⁻CD19⁺AnnexinV⁺PI⁻ early apoptotic B cells (f), and CD3⁻CD19⁺AnnexinV⁺PI⁺ late apoptotic B cells (g) in the spleen and dLNs of *Ythdf1*-deficient mice and WT control mice. **$p<0.01$. Two-way ANOVA with Sidak's multiple comparisons test.

Supplementary Fig. 4

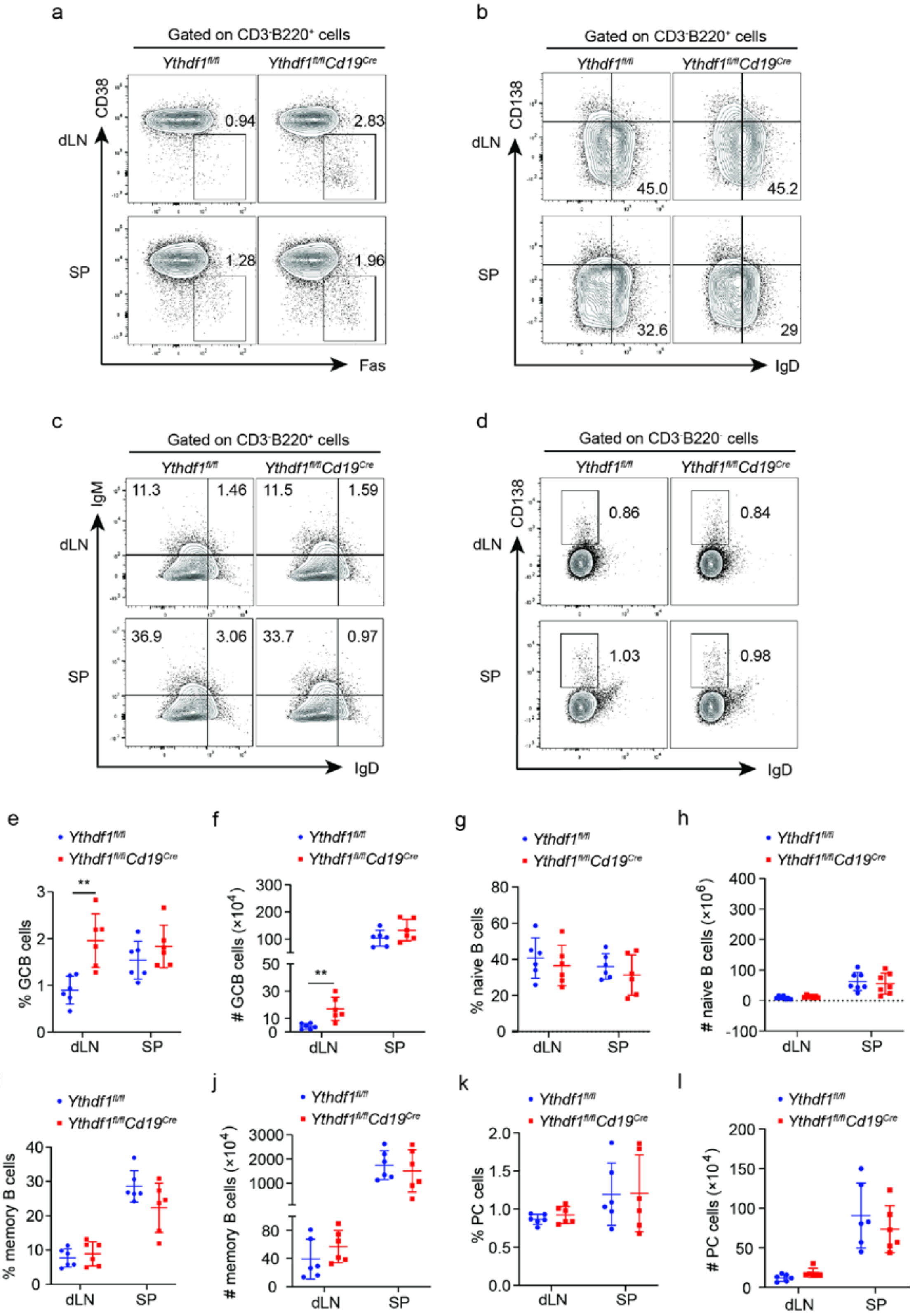

Supplementary Fig. 4 YTHDF1 is dispensable for the differentiation of naïve B, memory B, and PCs in mice in the steady state. a-d Representative dot plots showing the proportions of CD3$^-$B220$^+$ cells (a-c) and CD3$^-$B220$^-$ cells (d) in the spleen and dLNs of *Ythdf1*-deficient mice and WT control mice. **e-l** Quantification of the frequency and absolute number of CD3$^-$B220$^+$CD38$^-$Fas$^+$ GC B cells (e-f), CD3$^-$

B220^{+}IgD^{+}CD138^{-} naïve B cells (g-h), CD3^{-}B220^{+}IgD^{-}IgM^{+} memory B cells (i-j) , and CD3^{-}B220^{-}IgD^{-}CD138^{+} PCs (k-l) in the spleen and dLNs of *Ythdf1*-deficient mice and WT control mice, n=6. **p<0.01. Two-way ANOVA with Sidak's multiple comparisons test.

Supplementary Fig. 5

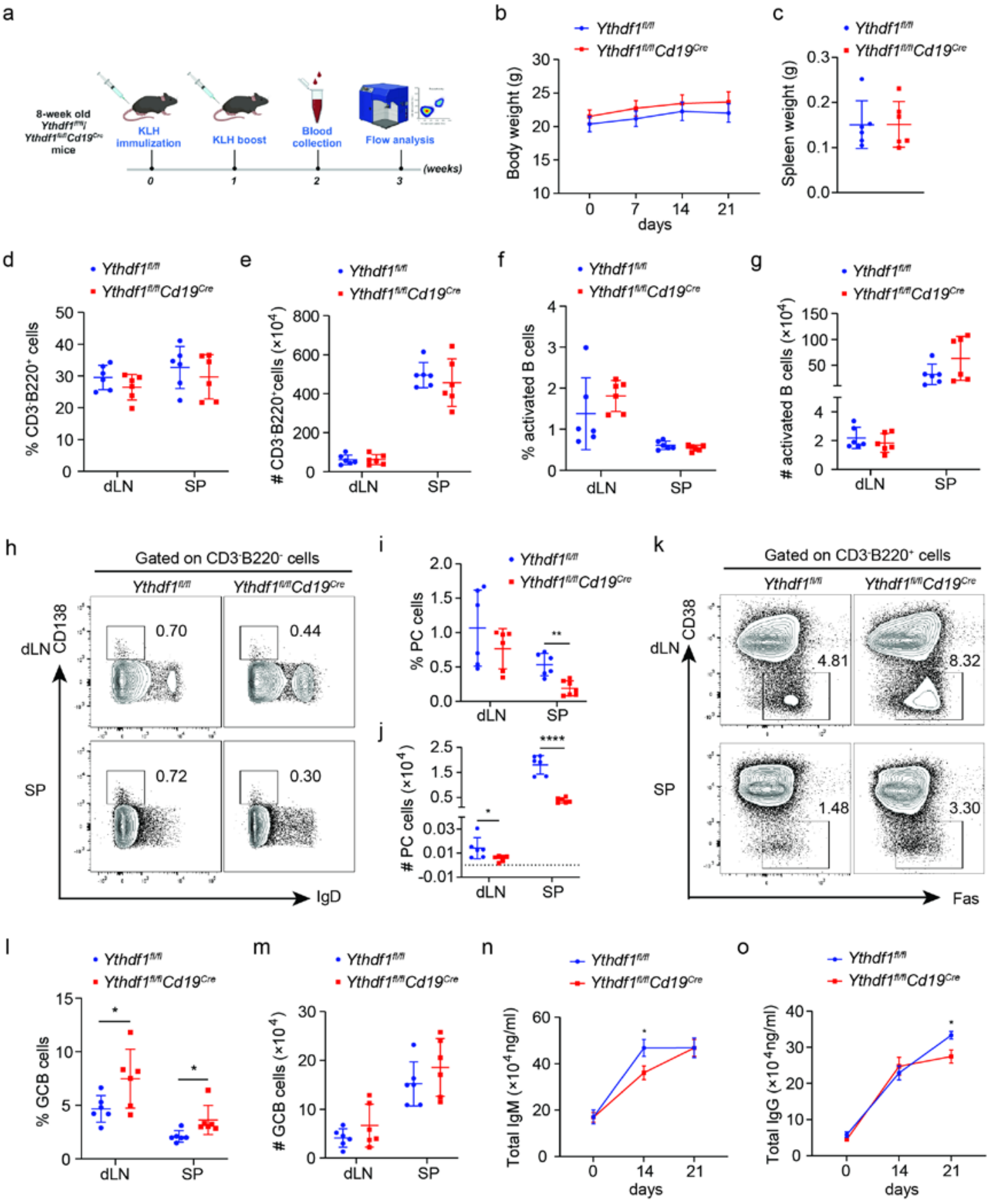

Supplementary Fig. 5 YTHDF1 depletion inhibits PC differentiation in KLH-immunized mice. a Schematic of the KLH-immunized mouse model. **b** The body weights of *Ythdf1*-dificient mice and WT control mice after KLH challenge, n=6. **c** The spleen weights of KLH-immunized *Ythdf1*-deficient mice and WT mice. **d-g** Quantification of the frequency and number of CD3$^-$B220$^+$ B cells (d-e) and CD3$^-$B220$^+$CD69$^+$ activated B cells (f-g) in the spleen and dLNs of *Ythdf1*-deficient mice and WT mice after KLH challenge. **h** Representative dot plots showing the proportions of CD3$^-$B220$^-$ cells in the spleen and dLNs of KLH-immunized *Ythdf1*-deficient mice and WT mice. **i-j** Quantification of the frequency (i) and absolute number (j) of CD3$^-$B220$^-$IgD$^-$CD138$^+$ PCs. **k** Representative dot plots showing the proportions of CD3$^-$B220$^+$ cells in the spleen and dLNs of KLH-immunized *Ythdf1*-deficient mice and WT mice. **l-m** Quantification of the frequency (l) and absolute number (m) of CD3$^-$B220$^+$CD38$^-$Fas$^+$ GC

B cells. **n-o** Total IgM (n) and IgG (o) antibodies in the serum of KLH-immunized *Ythdf1*-deficient mice and WT mice were determined by ELISA (mean ± SEM). *p<0.05, **p<0.01, ****p<0.0001. (b-c, n-o) Unpaired two-tailed Student's *t* test; (d-g, i-j, l-m) two-way ANOVA with Sidak's multiple comparisons test.

Supplementary Fig. 6

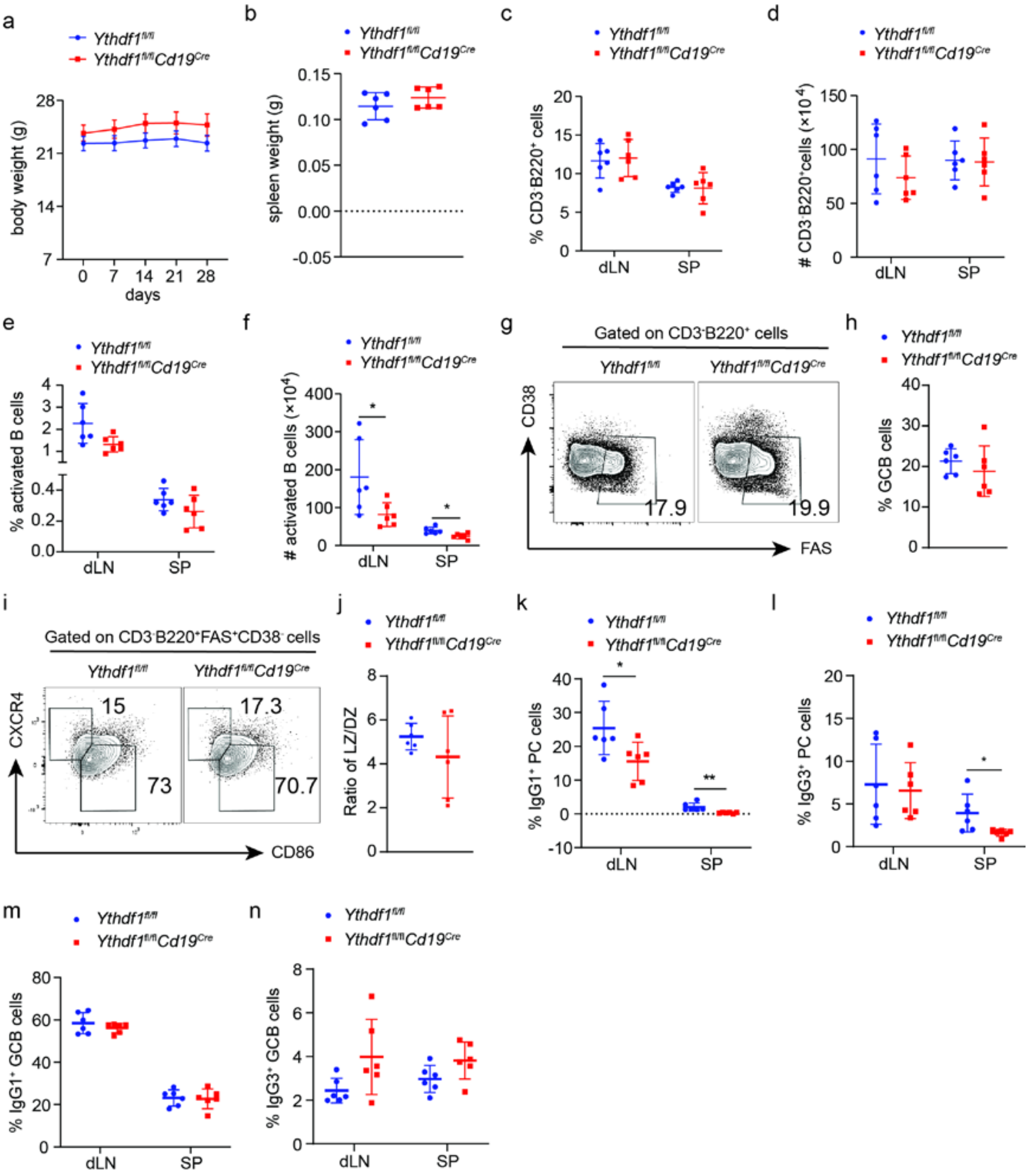

Supplementary Fig. 6 YTHDF1 depletion results in reduced absolute number of activated B cells in NP-KLH-immunized mice. a-b The weights of body (a) and spleen (b) of *Ythdf1*-deficient mice and WT control mice after NP-KLH challenge. **c-f** Quantification of the frequency and absolute number of $CD3^{-}B220^{+}$ B cells (c-d) and $CD3^{-}B220^{+}CD69^{+}$ activated B cells (e-f) in the spleen and dLNs of *Ythdf1*-deficient mice and WT control mice after NP-KLH challenge, n=6. **g** Representative dot plots showing the proportions of $CD3^{-}B220^{+}$ cells in the dLNs of np-KLH-immunized *Ythdf1*-deficient mice and WT mice. **h** Quantification of the frequency of $CD3^{-}B220^{+}Fas^{+}CD38^{-}$ GC B cells, n=6. **i** Representative dot plots showing the proportions of $CD3^{-}B220^{+}Fas^{+}CD38^{-}$ GC B cells in the dLNs of np-KLH-immunized *Ythdf1*-deficient mice and WT mice. **j** The ratio of LZ ($CD86^{hi}CXCR4^{lo}$) to DZ ($CD86^{lo}CXCR4^{hi}$) GCBs in the dLNs of NP-KLH-immunized Ythdf1-deficient mice and WT mice, n=6. **k-n** Quantification of $IgG1^{+}$ PCs (k), $IgG3^{+}$ PCs (l), $IgG1^{+}$ GCB cells (m) and $IgG3^{+}$ GCB cells (n), n=6. *p<0.05. (a-b, h, j-n)

Unpaired two-tailed Student's *t* test; (c-f) two-way ANOVA with Sidak's multiple comparisons test.

Supplementary Fig. 7

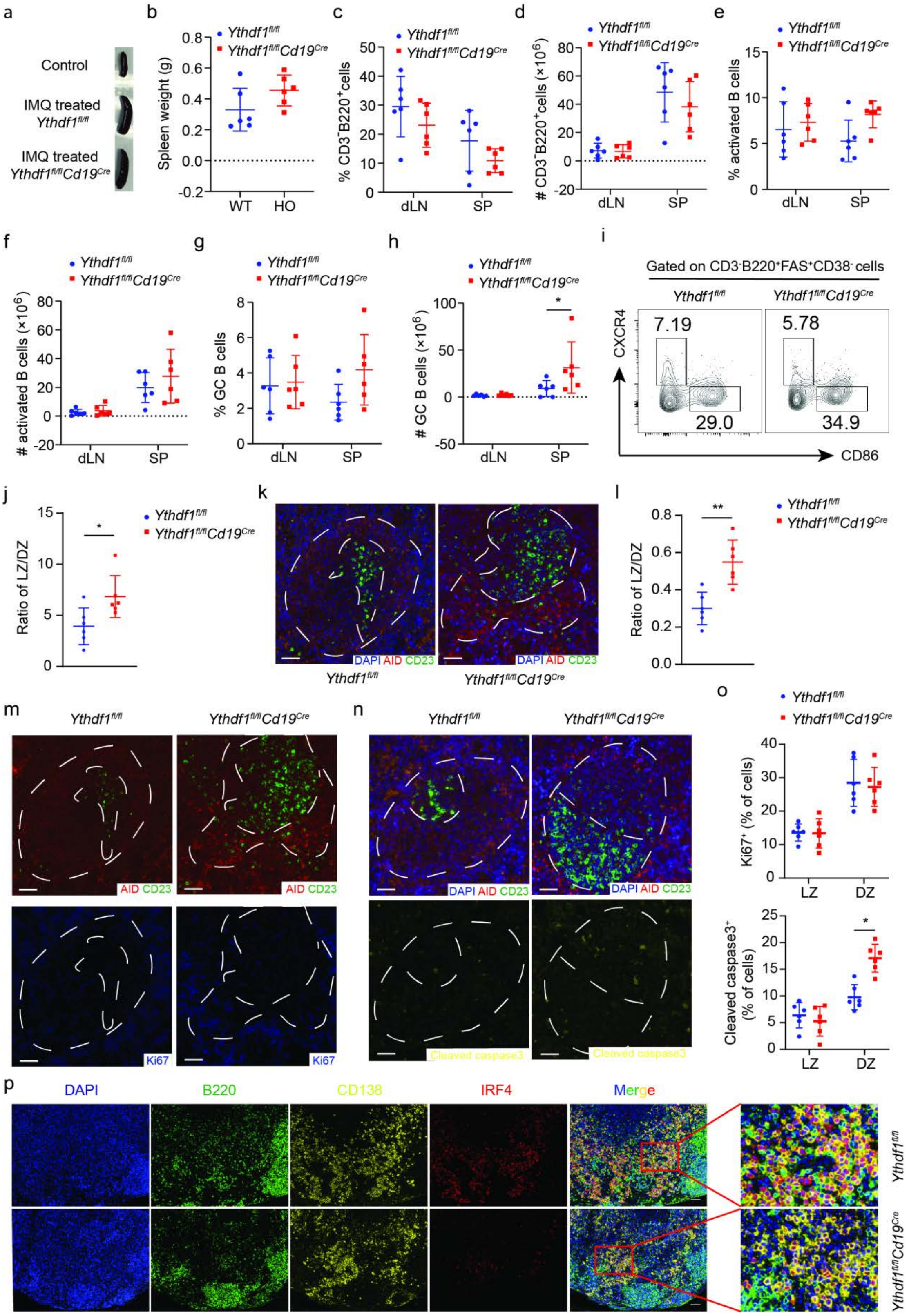

Supplementary Fig. 7 IRF4 expression is decreased in PCs in *Ythdf1*-deficient mice following IMQ treatment. a Representative spleen images of untreated WT, IMQ-treated WT, and IMQ-treated *Ythdf1*-

null mice. **b** Spleen weights of WT control and *Ythdf1*-null mice after IMQ treatment, n=6. **c-h** Quantification of the frequency and absolute number of CD3$^-$B220$^+$ B cells (c-d), CD3$^-$B220$^+$CD69$^+$ activated B cells (e-f), and CD3$^-$B220$^+$CD38$^-$Fas$^+$ GC B cells (g-h) in the spleen and dLNs of WT control and *Ythdf1*-null mice after IMQ treatment. **i** Representative dot plots showing the proportions of CD3$^-$B220$^+$Fas$^+$CD38$^-$ GC B cells in the spleen of IMQ-treated *Ythdf1*-deficient mice and WT mice. **j** The ratio of LZ (CD86^{hi}CXCR4^{lo}) to DZ (CD86^{lo}CXCR4^{hi}) GCBs in the spleen of IMQ-treated Ythdf1-deficient mice and WT mice, n=6. **k** Representative histological images showing LZ (CD23$^+$) and DZ (AID$^+$) GC B cells from IMQ-treated control and Ythdf1-null mice, scale bar: 100µm. **l** The ratio of LZ (CD23$^+$) to DZ (AID$^+$) GCBs of IMQ-treated Ythdf1-deficient mice and WT mice, n=6. **m** Representative histological images showing Ki67$^+$ LZ (CD23$^+$) and DZ (AID$^+$) GC B cells from IMQ-treated control and Ythdf1-null mice, scale bar: 100µm. **n** Representative histological images showing casepase3$^+$ LZ (CD23$^+$) and DZ (AID$^+$) GC B cells from IMQ-treated control and Ythdf1-null mice, scale bar: 100µm. **o** Quantification of the frequency of Ki67$^+$ LZ (CD23$^+$) and DZ (AID$^+$) GC B cells and casepase3$^+$ LZ (CD23$^+$) and DZ (AID$^+$) GC B cells from IMQ-treated control and Ythdf1-null mice, n=6. **p** Representative histological images showing IRF4 expression in PCs from IMQ-treated control and Ythdf1-null mice, scale bar: 50µm. *$p<0.05$, **$p<0.01$. (b, j, l) Unpaired two-tailed Student's *t* test; (c-h, o) two-way ANOVA with Sidak's multiple comparisons test.

Supplementary Fig. 8

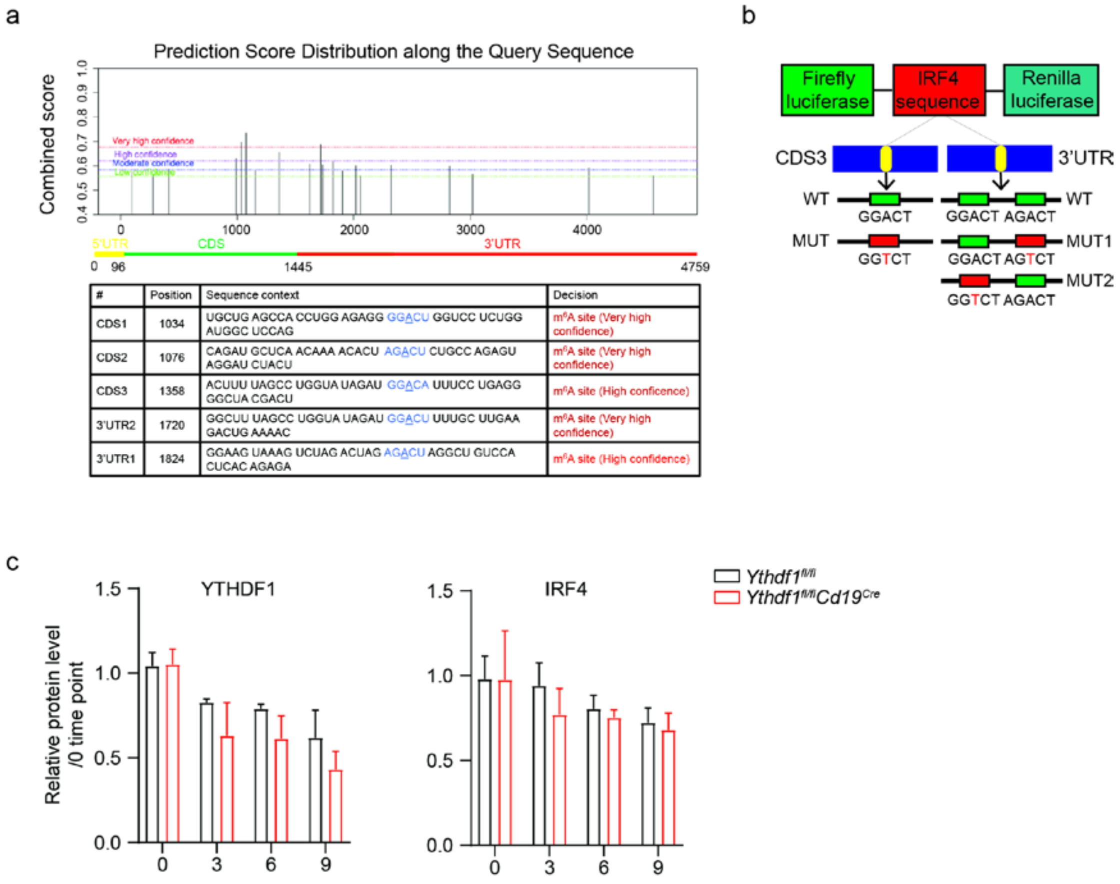

Supplementary Fig. 8 YTHDF1 binds to m6A-tagged 3'UTR of Irf4 mRNA. a The m^{6}A binding sites on *Irf4* mRNA with high confidence or very high confidence were predicted by SRAMP analysis.

b Schematic showing the construction of luciferase reporter vectors containing the *Irf4* coding sequence (CDS) or 3’ untranslated region (3’UTR) sequence. Adenosine was replaced by thymine to generate the mutation form of m^6A consensus sequence. **c** Quantification of YTHDF1 and IRF4 protein levels.

Supplementary Table 1. Information on HCs and patients

Identifier	Gender	Age	SLEDAI score
SLE1	Female	38	0
SLE2	Female	36	4
SLE3	Female	19	4
SLE4	Female	32	2
SLE5	Female	37	0
SLE6	Female	27	4
SLE7	Female	41	4
SLE8	Female	33	0
SLE9	Female	71	4
SLE10	Female	26	0
SLE11	Female	60	8
SLE12	Female	43	0
SLE13	Female	34	0
SLE14	Female	24	6
SLE15	Female	34	4
SLE16	Female	57	2
SLE17	Female	65	8
SLE18	Female	25	1
SLE19	Male	28	0
SLE20	Female	29	12
SLE21	Female	54	4
SLE22	Female	26	4
SLE23	Female	52	4
SLE24	Female	50	0
SLE25	Female	43	0
SLE26	Female	57	0
SLE27	Female	52	0
SLE28	Female	37	0
SLE29	Female	35	0
SLE30	Female	59	0
SLE31	Female	50	4
SLE32	Female	54	4
SLE33	Female	55	0
SLE34	Female	24	4
SLE35	Female	23	14
SLE36	Female	55	8
SLE37	Female	38	4
SLE38	Female	55	2

SLE39	Male	25	8
SLE40	Female	29	4
SLE41	Female	41	2
SLE42	Female	36	0
SLE43	Female	31	4
SLE44	Female	54	0
SLE45	Female	30	6
SLE46	Female	48	0
SLE47	Female	50	0
SLE48	Female	60	8
SLE49	Female	20	4
SLE50	Female	42	0
SLE51	Male	17	2
SLE52	Male	73	4
SLE53	Male	74	2
SLE54	Male	19	4
SLE55	Male	49	9
SLE56	Female	59	7
SLE57	Female	37	8
SLE58	Male	34	6
SLE59	Male	31	10
SLE60	Female	57	5
SLE61	Female	45	7
SLE62	Female	29	4
SLE63	Female	38	NA
SLE64	Male	70	NA
SLE65	Female	30	NA
SLE66	Female	37	NA
SLE67	Female	41	NA
SLE68	Female	28	NA
SLE69	Female	46	NA
SLE70	Female	61	NA
SLE71	Female	35	NA
SLE72	Male	25	NA
SLE73	Female	52	NA
SLE74	Female	48	NA
SLE75	Female	21	NA
SLE76	Female	41	NA
SLE77	Female	34	NA
SLE78	Female	54	NA
SLE79	Female	57	NA
SLE80	Female	37	NA

SLE81	Female	52	NA
SLE82	Female	42	NA
SLE83	Female	46	NA
SLE84	Female	34	NA
SLE85	Female	52	NA
SLE86	Female	32	NA
SLE87	Female	42	NA
SLE88	Female	38	NA
SLE89	Male	57	NA
SLE90	Female	30	NA
SLE91	Female	21	NA
SLE92	Male	29	NA
SLE93	Female	37	NA
SLE94	Female	47	NA
HC1	Female	69	NA
HC2	Female	31	NA
HC3	Female	39	NA
HC4	Female	30	NA
HC5	Female	37	NA
HC6	Female	56	NA
HC7	Female	50	NA
HC8	Female	54	NA
HC9	Female	65	NA
HC10	Female	23	NA
HC11	Female	37	NA
HC12	Female	55	NA
HC13	Female	53	NA
HC14	Female	35	NA
HC15	Female	51	NA
HC16	Female	54	NA
HC17	Female	45	NA
HC18	Female	27	NA
HC19	Female	64	NA
HC20	Female	28	NA
HC21	Female	60	NA
HC22	Female	67	NA
HC23	Female	17	NA
HC24	Female	39	NA
HC25	Female	72	NA
HC26	Female	65	NA
HC27	Female	22	NA
HC28	Female	51	NA

HC29	Female	29	NA
HC30	Male	33	NA
HC31	Male	22	NA
HC32	Female	51	NA
HC33	Female	49	NA
HC34	Male	65	NA
HC35	Female	38	NA
HC36	Female	47	NA
HC37	Female	37	NA
HC38	Female	54	NA
HC39	Female	46	NA
HC40	Female	54	NA
HC41	Female	54	NA

SLE1-SLE58 were recruited for comparing YTHDF1 expression in peripheral B cell subsets and analyzing the correlation between YTHDF1 expression in PCs and SLEDAI score, complement C3, C4, anti-dsDNA antibody, ANA, anti-smith antibody, and proteinuria, respectively. HC1-41 were recruited for comparing YTHDF1 expression in peripheral B cell subsets. SLE59-SLE94 were enrolled for the correlation analysis of YTHDF1 expression and IRF4 expression in peripheral PCs. SLE: Systemic lupus erythematosus; SLEDAI: SLE disease activity index; HC: healthy control; NA: not applicable.

Supplementary Table 2. Sequence information on siRNA and primers

Target	Sequence
Mouse-Tet2	Forward: 5’-3’ TTCCTGTTCCGCTACGGTG
	Reverse:5’-3’ GGTGGTTCTGTCCTGTTCCA
Mouse-Irf4	Forward:5’-3’ AAGACGTTCGGTCAGCTCTCCA
	Reverse:5’-3’ CTGGCACTCATGTGGCTTCTCT
Mouse-Ern1	Forward:5’-3’ ATGTGGCCCTGAAACCTTGT
	Reverse:5’-3’ AAGTGGGAGCAAGCTCATCC
Mouse-Saraf	Forward:5’-3’ GCACCAGGGCTTCTCTGATT
	Reverse:5’-3’ AGAGCTTGCACCATAGCCAG
Mouse-Lef1	Forward:5’-3’ TGCGATCCTCCAGCATCTTC
	Reverse:5’-3’ ACAGGAGCGAGAGGAGTCTT
Mouse-GAPDH	Forward:5’-3’ TGGAAAGCTGTGGCGTGAT
	Reverse:5’-3’ ACACATTGGGGGTAGGAACAC
Mouse-YTHDF1	Forward:5’-3’ CGCCATCTGGAGTTGGAGTT
	Reverse:5’-3’ GCGTGCAGATTCAGTATGCG
Mouse-Neat1	Forward:5’-3’ GTTGCTGATAGCGAAGCGTG
	Reverse:5’-3’ CCTTCTGAGGCCTGTTCCAG
Human-TET2	Forward:5’-3’ GATAGAACCAACCATGTTGAGGG
	Reverse:5’-3’ TGGAGCTTTGTAGCCAGAGGT
Human-ERN1	Forward: 5’-3’ ACCAGCGTGGTGATAGTTGG
	Reverse:5’-3’ CTGGAATTGCCGGTCCTTCT
Human-IRF4	Forward:5’-3’ ACCGAAGCTGGAGGGACTAC
	Reverse:5’-3’ GTGGGGCACAAGCATAAAAG
Human-SARAF	Forward:5’-3’ ACAGAATGTTGCTGCGGGAT
	Reverse:5’-3’ TAGCCTTCACAGCTCACCAC
Human-LEF1	Forward:5’-3’ TGCATCAGGTACAGGTCCAAG
	Reverse:5’-3’ ACGTTGGGAATGAGCTTCGT
Human-GAPDH	Forward: 5’-3’ GGAGCGAGATCCCTCCAAAAT
	Reverse:5’-3’ GGCTGTTGTCATACTTCTCATGG
Human-YTHDF1	Forward: 5’-3’ TTGTTCATGAAGCATGTCGGC
	Reverse: 5’-3’ AGTAGACCACGGAGCCTCAT
YTHDF1 siRNA pool	1#Sense: 5’-3’ ACGGCAGAGTCGAAACAAA
	1#Antisense: 5’-3’ TTTGTTTCGACTCTGCCGT
	2#Sense: 5’-3’ CTCCACCCATAAAGCATAA
	2#Antisense: 5’-3’ TTATGCTTTATGGGTGGAG
	#3Sense: 5’-3’ GCCGTCCATTGGATTTCCT
	#3Antisense: 5’-3’ AGGAAATCCAATGGACGGC
MsIRF4 CDS1#	Forward: 5’-3’ GAACATTGAGAAGTTGCTGAGCC
	Reverse: 5’-3’ ATAAAGCCCATCTGGAGCCATC
MsIRF4 CDS2#	Forward: 5’-3’ GGGACTGGTCCTCTGGATGG
	Reverse: 5’-3’ CCCAGTAGATCCTACTCTGGC

MsIRF4 CDS3#	Forward: 5’-3’ TGGCTTTAGCCTGGTATAGATGG
	Reverse: 5’-3’ TCTTGTCAGGGATTCATGCTGG
MsIRF4 3’UTR1#	Forward: 5’-3’ TTGGTCCATTTCTCAGGGAAGTA
	Reverse: 5’-3’ GCTCCTCTATCCAGGAATGCG
MsIRF4 3’UTR2#	Forward: 5’-3’ TGTGAAACTTGATCAGTGTTGTGT
	Reverse: 5’-3’ GATTCATGCTGGCACAGGTT

Supplementary Table 3. Information on antibodies for western blot, ELISA, and histology

Target	Source	Dilution
β-actin	Proteintech, 66009-1-Ig	1:1000
GAPDH	Proteintech, 60004-1-Ig	1:1000
YTHDF1	Proteintech,17479-1-AP	1:1000
IRF4	Proteintech,11247-2-AP	1:10000
IgM	Bethyl Laboratories, A90-101P	1:3000
IgG1	Bethyl Laboratories, A90-105P	1:3000
IgG2a	Bethyl Laboratories, A90-107P	1:3000
IgG2b	Bethyl Laboratories, A90-109P	1:5000
IgG (H+L)	Bethyl Laboratories, A90-116P	1:3000
IgG3	Bethyl Laboratories, A90-111P	1:3000
C3	Abcam, ab200999	1:1000
IgG	Proteintech, SA00001-2	1:1000
CD23	abcam, ab315289	1:2000
AID	invitrogen, 392500	1:20
Ki67	cell signaling, 9027S	1:1000
cleaved caspase3	CST, 9661	1:1000

Supplementary Table 4. Reagents for flow cytometry analysis

Reagent	Source	Identifier
FITC anti Hu/Mo IRF4	eBioscience	11-9858-82
Cytofix/Cytoperm^{TM} Fixation/Permeabilization Solution Kit	BD Pharmingen	554714
Zombie NIR^{TM} Fixable Viability Kit	Biolegend	423106
PE-Cyanine7 Mouse Anti-Human CD27	eBioscience	25-0279-42
APC Anti-Human CD19	Biolegend	392504
APC-Cy^{TM}7 Mouse Anti-Human CD20	BD Pharmingen	335794
PerCP-Cy^{TM}5.5 Mouse Anti-Human IgD	BD Pharmingen	561315
PE Mouse Anti-Human CD38	BD Pharmingen	555460
APC Anti-Human CD69	Biolegend	310910
FITC Rat Anti-Mouse CD3	BD Pharmingen	555274
PE/Cyanine7 Anti-Mouse CD69	Biolegend	104512
APC-CY^{TM}7 Hamster Anti-Mouse CD69	BD Pharmingen	8249672
APC Rat Anti-Mouse CD45R/B220	BD Pharmingen	553092
FITC Anti-Mouse/Human CD45R/B220	Biolegend	103206
PE Hamster Anti-Mouse CD95	BD Pharmingen	554258
APC Anti-Mouse CD38	Biolegend	102712
PerCP-Cy^{TM}5.5 Hamster Anti-Mouse CD3e	BD Pharmingen	551163
PE-Cy^{TM}7 Rat Anti-Mouse CD45R/B220	BD Pharmingen	552772
APC Rat Anti-Mouse CD138	BD Pharmingen	558626
PE/Cyanine7 Anti-Mouse CD138	Biolegend	142514
FITC Anti-Mouse IgD	Biolegend	405704
PE Rat Anti-Mouse IgM	BD Pharmingen	553409
PE Anti-Mouse/Human Ki67	eBioscience	12-5698-82
PerCP-Cy^{TM}5.5 Anti-Mouse CD38	BD Pharmingen	551400
APC/Cyanine7 Anti-Mouse IgD	Biolegend	405716
APC-Cy^{TM}7 Rat Anti-Mouse CD3	BD Pharmingen	560590
APC Anti-Mouse CD19	Biolegend	152409
APC/Cyanine7 Anti-Mouse CD19	Biolegend	115530
FcR Blocking Reagent, mouse	Miltenyi Biotec	130-092-575
PerCP-Cy^{TM}5.5 Anti-Mouse CXCR4	Biolegend	146507
PE Anti-Mouse CD86	Biolegend	105007
